# Isolation and characterization of *Trichoderma* from Amazonian white-water river sediments with descriptions of five new species and new records from Brazil

**DOI:** 10.3389/fmicb.2026.1778622

**Published:** 2026-05-20

**Authors:** Thiago Fernandes Sousa, Raoni Gwinner, Ingride Jarline Santos da Silva, Gleucinei dos Santos Castro, Gerodes Vasconcelos da Costa, Marta Cristina Corsi de Filippi, Candido Athayde Sobrinho, Michel Eduardo Beleza Yamagishi, Rogério Eiji Hanada, Aleksander Westphal Muniz, Felipe Ribeiro Fayad, Hector Henrique Ferreira Koolen, Gilvan Ferreira da Silva

**Affiliations:** 1 Embrapa Amazônia Ocidental, Manaus, Brazil; 2 Programa de Pós-Graduação em Biodiversidade e Biotecnologia, Universidade do Estado do Amazonas (UEA), Manaus, Brazil; 3 Programa de Pós-Graduação em Genética e Biologia Molecular, Universidade Federal do Rio Grande do Sul, Porto Alegre, Brazil; 4 Embrapa Arroz e Feijão, Santo Antônio de Goiás, Brazil; 5 Embrapa Meio Norte, Teresina, Brazil; 6 Embrapa Agricultura Digital, Campinas, Brazil; 7 Programa de Pós-Graduação em Agricultura nos Trópicos Húmidos, Instituto de Pesquisas da Amazônia (INPA), Manaus, Brazil; 8 Pontifícia Universidade Católica de Goiás (PUC-GO), Goiânia, Brazil; 9 Universidade do Estado do Amazonas (UEA), Manaus, Brazil

**Keywords:** biocontrol agents, biosynthetic gene clusters, CAZymes, *Colletotrichum*, mycoparasitism, phylogenomics, secondary metabolites

## Abstract

*Trichoderma* is a cosmopolitan genus widely used in agriculture due to its beneficial traits that promote plant growth and provide sustainable crop protection. Over 450 valid *Trichoderma* species have been described, with increasing whole-genome data showing extensive enzymatic repertoires and biosynthetic potential. However, the Amazonian biome remains underexplored for *Trichoderma* diversity despite its megadiversity. In this study, 44 *Trichoderma* strains were isolated from sediments collected during a systematic expedition along three major white-water Amazonian rivers (Juruá, Madeira, and Purus). Initial molecular barcoding using *tef1-α* sequences identified 14 species, with *Trichoderma lentiforme* being ubiquitously distributed across all three river systems. Isolates exhibiting <97% sequence identity to known species underwent comprehensive polyphasic characterization, including multi-locus phylogenetic analyses (*tef1-α* and *rpb2*), morphological studies, biocontrol assays, and comparative genomics. This approach led to the description of five novel species (*Trichoderma madeiraense*, *Trichoderma labreaense*, *Trichoderma tapauaense*, *Trichoderma submersum*, and *Trichoderma juburiaense*). It established the first records of *Trichoderma cyanodichotomus* and *Trichoderma awajun* for Brazil. All newly described species demonstrated strong antagonistic activity against major phytopathogens, with *T. juburiaense* TM67 achieving >95% mycelial growth inhibition against multiple *Colletotrichum* species. Comparative genomic analyses revealed diverse CAZyme arsenals enriched in glycoside hydrolases associated with mycoparasitism (GH16, GH18, GH72) and lignocellulose degradation, with *T. labreaense* TM26 exhibiting particularly high chitinase content and *T. juburiaense* TM67 showing elevated β-glucanase levels. Genome mining identified biosynthetic gene clusters encoding bioactive secondary metabolites, including verticillins, ilicicolins, chaetoglobosins, harzianopyridone, and brefeldin A, compounds with established antifungal, antibacterial, and cytotoxic activities. This study provides a taxonomic and genomic survey of *Trichoderma* from Amazonian white-water river sediments, describing five novel species and highlighting this frontier ecosystem as an important but undersampled source of fungal biodiversity.

## 1 Introduction

The genus *Trichoderma* has a cosmopolitan distribution across diverse environments and is predominantly found in plant tissues, decaying wood, sediments, and different soil types (Woo et al., 2022). Species within this genus exhibit notable physiological traits that are beneficial for biotechnology and are widely used in industry and agriculture due to their diverse enzyme repertoires combined with high-titer production, biosynthesis of bioactive secondary metabolites, a genetic toolbox, and mycoparasite activity (Bischof and Seiboth, 2014; Baccelli et al., 2022; Woo et al., 2022).

Many species of *Trichoderma* have been described in recent years, driven by molecular data and evolutionary analysis, which enabled the description of 375 species with valid nomenclature as of 2020 (Woo et al., 2022). A comprehensive guideline for *Trichoderma* identification proposed that the DNA barcodes ITS, *tef1*-*α*, and *rpb2* can be used for unambiguous identification and species delimitation, establishing that >70% similarity in ITS sequence data confirms the genus, while nucleotide identity <99% in *rpb2* and <97% in *tef1*-α relative to type isolates supports new species status when combined with phylogenetic and morphological analysis (Cai and Druzhinina, 2021). Data from MycoBank^1^ indicate that since 2021, an additional 114 species have been formally described through November 19, 2025. When combined with the 375 species recognized in Woo et al.’s (2022) revision, this brings the total number of *Trichoderma* species with valid nomenclature to 489.

Despite *Trichoderma* presenting many species and well-characterized strains, the search for these microorganisms continues to grow. New potent biocontrol strains that control phytopathogens and improve crop production with economic impacts are within the scope of *Trichoderma* studies (Ghazanfar et al., 2018; Abdullah et al., 2021). Brazil is the second-largest producer of agricultural products and has a megadiverse biodiversity and multiple ecosystems that have been little explored for microbial diversity (Pylro et al., 2014; FAO and OECD, 2015; Oliveira et al., 2019). Studies on *Trichoderma* in the Amazon resulted in the discovery of phosphorus-solubilizing and plant growth-promoting strains, as well as new species (Holmes et al., 2004; Samuels et al., 2006a,b; López-Quintero et al., 2013; Bononi et al., 2020; Sousa et al., 2023). A comprehensive study of *Trichoderma* diversity in the Bolivian Amazon identified 51 different species, including 41 isolates representing putative new species (Mamani et al., 2025).

The different ecosystems of the Amazon rainforest are highly interconnected in a complex network of interactions, in which the huge amount of plant biomass is maintained by an intense activity of saprophytic microorganisms, which is enhanced by the hot and humid climate of the region. These microorganisms recycle carbon by decomposing countless layers of organic material in the soil, thus returning to the forest the nutrients necessary for its maintenance (Malhi, 2012). The product of decomposition is leached by heavy rainfall, deposited in rivers, and spread throughout the biome via flood pulses, accumulating in sediments (Junk, 2013; Junk et al., 2015; de Sousa Lobo et al., 2019; Melack et al., 2021).

These rivers are classified into three main water types based on physicochemical properties: white-water, black-water, and clear-water. White-water rivers, including the Madeira, Juruá, and Purus, originate in the Andes and transport large quantities of nutrient-rich suspended sediments with near-neutral pH and elevated electrical conductivity (McClain and Naiman, 2008). The Madeira River alone may contribute up to 50% of the Amazon’s total sediment discharge to the Atlantic Ocean (Vauchel et al., 2017). River sediments represent heterogeneous microbial environments containing mixtures of organic material at various stages of decomposition, creating diverse niches that select for fungi with broad enzymatic capabilities for lignocellulose degradation (Dunne et al., 1998). Recent metagenomic analyses revealed that Amazonian river microbiomes harbor substantial genetic novelty compared to temperate river systems (Santos-Júnior et al., 2020), suggesting white-water sediments as underexplored sources of microbial biodiversity.

In this study, we explored Amazonian river systems and collected sediment samples to isolate and characterize *Trichoderma* strains. A total of 44 isolates were phylogenetically identified to species level through *tef1*-*α* sequence analysis. Among these, five isolates represented putatively novel species. They were subjected to comprehensive polyphasic characterization, including detailed morphological descriptions, multi-locus phylogenetic analysis (*tef1*-α and *rpb2*), CAZyme profiling, biosynthetic gene cluster prediction, and evaluation of antagonistic activity against various phytopathogenic species using dual-culture assays.

## 2 Materials and methods

### 2.1 Isolation and culture conditions

**Figure 1:**
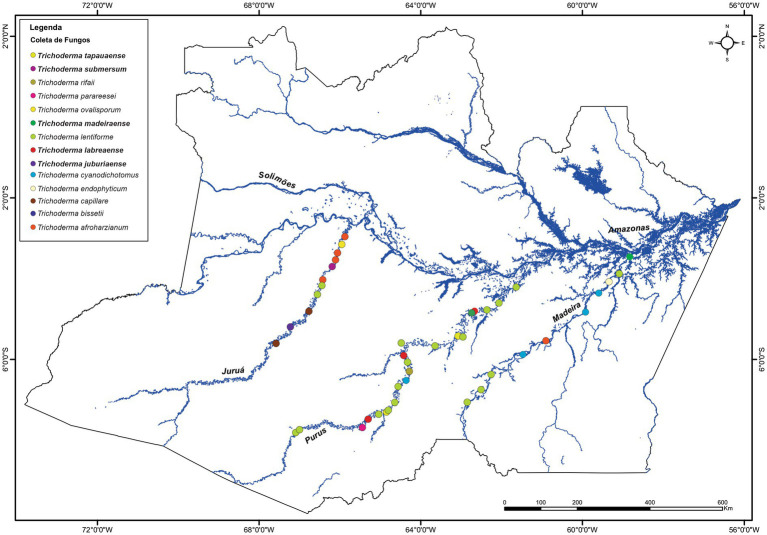
Geographical distribution of *Trichoderma* isolates across the Madeira, Juruá, and Purus river systems in Amazonas State, Brazil. Collection sites are indicated by colored circles corresponding to species identity as determined by *tef1-α* phylogenetic analysis. The map shows the major river network of the Amazon Basin with isolates distributed along multiple sampling points. Scale bar represents distance in kilometers.

The *Trichoderma* isolates were obtained from sediments of different Amazonian rivers located in the state of Amazonas (Brazil). The exact geographic coordinates of sediment collection sites for each strain, along with sediment depth and water pH, are shown in Table 1 and Figure 1.

**Table 1:** *Trichoderma* isolates from Amazonian river sediments with corresponding species identification, geographical origin, and physicochemical parameters.

Isolate	*tef1-α*	Species	Geographical location	River	Municipality	RD	WT (°C)	WpH
TM2	PV695505	*T. lentiforme*	07°03′12.7 S 62°51′00.9 W	Madeira	Humaitá	4.0	29.0	6.0
TM3	PV695506	*T. lentiforme*	06°44′40.9 S 062°30′35. W	Madeira	Humaitá	3.5	26.0	6.0
TM4	PV695507	*T. lentiforme*	06°21′51.4 S 062°15′31.5 W	Madeira	Humaitá	1.0	31.5	6.0
TM5	PV695508	*T. cyanodichotomus*	06°12′13.0 S 061°50′10.6 W	Madeira	Humaitá	1.5	29.5	6.0
TM6	PV695509	*T. cyanodichotomus*	05°49′22.4 S 061°18′10.5 W	Madeira	Manicoré	0.5	27.0	6.0
TM7	PV695510	*T. afroharzianum*	05°31′56. S 060°54′12.0 W	Madeira	Manicoré	0.4	26.0	6.0
TM8	PV695511	*T. cyanodichotomus*	05°14′40.0 S 060°32′51.5 W	Madeira	Novo Aripuanã	1.0	24.0	6.0
TM11	PV695512	*T. cyanodichotomus*	04°23′11.8 S 059°35′43.1 W	Madeira	Borba	0.70	30.0	6.0
TM12	PV695513	*T. endophyticum*	04°04′11.1 S 059°20′52.0 W	Madeira	Borba	3.0	31.0	6.0
TM13	PV695514	*T. lentiforme*	03°52′24.0 S 059°05′25.4 W	Madeira	Nova Olinda do Norte	1.0	32.5	6.0
TM14*	PV695515	*T. madeiraense*	03°27′09.9 S 058°49′59.0 W	Madeira	Itacoatiara	1.0	30.5	6.0
TM18	PV695516	*T. lentiforme*	07°48′07.9 S 067°05′39.8 W	Purus	Pauini	1.0	29.0	6.0
TM19	PV695517	*T. lentiforme*	07°44′02.7 S 067°00′07.1 W	Purus	Pauini	0.40	29.0	6.0
TM25	PV695518	*T. parareesei*	07°38′48.3 S 065°28′03.4 W	Purus	Lábrea	1.5	25.0	6.0
TM26*	PV695519	*T. labreaense*	07°28′22.9 S 065°18′10.1 W	Purus	Lábrea	0.30	25.0	6.0
TM27	PV695520	*T. lentiforme*	07°21′02.2 S 065°02′25.8 W	Purus	Lábrea	1.0	24.0	6.0
TM28	PV695521	*T. tapauaense*	07°17′35.8 S 064°51′02.7 W	Purus	Lábrea	2.0	24.0	6.0
TM29	PV695522	*T. lentiforme*	07°15′09.0 S 064°48′27.1 W	Purus	Lábrea	7.0	25.0	6.0
TM30	PV695523	*T. lentiforme*	07°03′28.8 S 064°38′48.1 W	Purus	Lábrea	5.0	26.0	5.5
TM32	PV695524	*T. lentiforme*	06°39′53.5 S 064°33′36.8 W	Purus	Canutama	6.5	32.0	5.5
TM34	PV695525	*T. rifaii*	06°17′22.0 S 064°17′12.6 W	Purus	Canutama	8.5	25.0	5.5
TM35	PV695526	*T. lentiforme*	06°03′45.3 S 064°19′55.0 W	Purus	Tapauá	4.5	24.0	5.5
TM36	PV695527	*T. labreaense*	05°53′45.6 S 064°25′29.7 W	Purus	Tapauá	4.0	24.0	5.5
TM39	PV695528	*T. lentiforme*	05°35′05.3 S 063°50′50.1 W	Purus	Tapauá	5.5	29.0	5.5
TM40	PV695529	*T. lentiforme*	05°39′32.2 S 063°38′53.9 W	Purus	Tapauá	4.5	27.0	5.5
TM42*	PV695530	*T. tapauaense*	05°24′56.2 S 063°04′55.1 W	Purus	Tapauá	4.0	29.0	5.5
TM43	PV695531	*T. lentiforme*	05°26′14.9 S 062°57′24.4 W	Purus	Tapauá	5.0	28.0	5.5
TM46	PV695533	*T. labreaense*	04°47′42.8 S 062°40′18.0 W	Purus	Beruri	6.0	26.0	5.5
TM47	PV695534	*T. lentiforme*	04°46′01.7 S 062°21′53.2 W	Purus	Beruri	5.0	31.0	5.5
TM48	PV695535	*T. lentiforme*	04°35′41.1 S 062°04′00.8 W	Purus	Beruri	7.5	29.0	5.5
TM49	PV695536	*T. cyanodichotomus*	04°24′29.6 S 061°56′16.5 W	Purus	Beruri	10.0	26.0	5.5
TM50	PV695537	*T. lentiforme*	04°12′16.8 S 061°38′30.2 W	Purus	Beruri	6.0	27.0	5.5
TM54	PV695538	*T. afroharzianum*	02°57′14.2 S 065°52′36.9 W	Juruá	Juruá	5.5	27.0	5.5
TM55	PV695539	*T. ovalisporum*	03°08′50.1 S 065°57′29.5 W	Juruá	Juruá	2.0	27.0	5.5
TM56	PV695540	*T. afroharzianum*	03°21′18.6 S 066°03′43.6 W	Juruá	Juruá	6.0	28.0	5.5
TM57	PV695541	*T. afroharzianum*	03°31′38.1 S 066°06′40.8 W	Juruá	Juruá	4.0	28.0	5.5
TM58*	PV695542	*T. submersum*	03°41′36.4 S 066°11′33.9 W	Juruá	Juruá	3.5	27.0	5.5
TM60	PV695543	*T. afroharzianum*	04°01′10.0 S 066°25′31.9 W	Juruá	Juruá	5.0	26.0	5.5
TM61	PV695544	*T. lentiforme*	04°10′02.1 S 066°26′56.7 W	Juruá	Juruá	6.5	26.0	5.5
TM62	PV695545	*T. lentiforme*	04°23′10.1 S 066°33′27.3 W	Juruá	Carauari	2.0	24.0	5.5
TM64	PV695546	*T. capillare*	04°48′05.9 S 066°46′00.3 W	Juruá	Carauari	4.5	26.0	5.5
TM67*	PV695547	*T. juburiaense*	05°11′20.4 S 067°13′41.8 W	Juruá	Carauari	2.0	24.0	5.5
TM70	PV695548	*T. awajun*	05°36′00.3 S 067°34′57.5 W	Juruá	Carauari	5.0	27.0	5.5
TM71	PV695549	*T. capillare*	05°36′00.8 S 067°34′58.7 W	Juruá	Carauari	3.0	27.0	5.5

RD, river depth (m); WT, water temperature (°C); WpH, water pH. Asterisk (*) indicates novel species described in this study. GenBank accession numbers correspond to *tef1-α* sequences.

The sediment samples were recovered from the bottom of the Amazon rivers using a Van Veen grab sampler and stored in Falcon tubes (50 mL) at 4 °C. The samples were dried under reduced pressure to obtain sediment pellets, from which a portion (1.0 g) was diluted to 10^−3^. A 100 μL volume of this solution was inoculated into Rose Bengal agar medium (1.0 g KH_2_PO_4_, 0.5 g MgSO4∙7H2O, 5.0 g peptone, 10 g dextrose, 0.35 g rose Bengal) and incubated at 30 °C for 4 days. Colonies with morphological characteristics of *Trichoderma* were picked and purified by monosporic culture, then preserved in potato dextrose broth medium (PD; 200 g potato, 20 g dextrose) and glycerol (1:1, v/v) at −80 °C.

### 2.2 DNA extraction, PCR, and sequencing

The strains were cultivated on PDA at 30 °C for 5 days to induce massive conidiation. A spore solution was prepared at a concentration of 10^8^, and 100 μL of this solution was inoculated into the PD medium and incubated at 30 °C with orbital shaking (150 rpm) for 2 days. The broth was filtered, and the obtained mycelial mass was dried with a sterile paper towel. The mycelia were then lysed in liquid nitrogen, and DNA extraction was performed using the 2% CTAB method (Doyle and Doyle, 1987). The DNA purity ratio (260/280 nm) was determined using a Nanodrop (Thermo Fisher), and the DNA integrity was assessed by gel electrophoresis (0.8% agarose). DNA was diluted to 50 ng/μL for the PCR reactions.

The PCR reactions were prepared for three primer pairs in which the internal transcribed spacer 1 (ITS), 5.8S, and ITS2 regions (primers: ITS1 and ITS4), the partial sequence of the gene *tef1-α* (primers: EF-1αF and EF-1αR), and the partial sequence of *the rpb2* gene (primers: fRPB2-5F and fRPB2-7cR) using the *EasyTaq*^®^ kit (Sinapse Biotecnologia) using the manufacturer’s recommendations. The PCR conditions are as follows: initial denaturation at 95 °C for 3 min; 35 cycles of denaturation at 95 °C for 15 s, annealing at primer-specific temperatures (Table 2) for 30 s, extension at 72 °C for 1 min, and final extension at 72 °C for 5 min.

**Table 2:** Specifications of primers and annealing temperatures used to amplify fragments.

Primer	Sequence	Annealing temperature °C	References
ITS1	TCCGTAGGTGAACCTGCGG	55	White et al. (1990)
ITS4	TCCTCCGCTTATTGATATG		
fRPB2-5F	GAYGAYMGWGATCAYTTYGG	58	Liu et al. (1999)
fRPB2-7cR	CCCATRGCTTGYTTRCCCAT		
EF-1αF	ATGGGTAAGGARGACAAGAC	57	O’Donnell et al. (1998) and Carbone and Kohn (1999)
EF-1αR	GGARGTACCAGTSATCATGTT		

To confirm that the target sequences were amplified and the absence of nonspecific amplification, the PCR products were resolved in agarose gel (Sigma: 1.5 g/100 mL) stained with ethidium bromide (0.05 μL/mL) and then photographed under UV light on a Molecular Imaging System equipment (Loccus Biotecnologic L-Pix. Chemi) and compared with a 1-kb ladder (Invitrogen).

The PCR purification was performed using exoSAP-IT (Thermo Fisher; catalog number: 78200.200. UL), according to the manufacturer’s recommendations. Sequencing reactions were performed in a volume of 10 μL, containing 2 μL of ultrapure water, 1.5 μL of BigDye buffer, 0.5 μL of BigDye terminator v3.1 (Thermo Fisher), 1 μL of each primer, and 5 μL of the purified PCR products. The cycling conditions were 96 °C for 1 min, followed by 35 cycles at 96 °C for 15 s, 50 °C for 15 s, and 60 °C for 4 min. Sequencing was performed using a genetic analyzer (3,500, Thermo Fisher).

### 2.3 Phylogenetic analysis, distance calculation, and SNP analysis

Consensus sequences of partial sequences of the ITS region, *tef1-α*, and *rpb2* were obtained manually based on the alignment of forward and reverse sequences as well as electropherogram analysis. The *tef1-α* sequences of 44 isolates obtained in this study were aligned with 332 sequences from the type species *Trichoderma* for preliminary identification by phylogenetic analysis. The isolates that showed indications of new species were subjected to a new analysis using concatenated *tef1-α* and *rpb2* sequences with a dataset containing closely related species.

The sequences for each locus were aligned individually using the MAFFT algorithm in UGENE software (Okonechnikov et al., 2012). The *tef1-α* and concatenated (*tef1-α* + *rpb2*) alignments were plotted in the IQTREE platform (Trifinopoulos et al., 2016) for the maximum likelihood (ML) analysis using 1,000 replicates (bootstraps).

Bayesian Inference (BI) analysis was based on the model adopted in PAUP* 4 and MrModeltest2 v2 (Nylander, 2004). All sites in the loci were considered, and the analysis was performed for 10 million generations, with the first 25% of trees discarded and burned using the MrBayes v 3.7 tool available from CIPRES^2^.

The branch supports and tree topology were visualized in the iTOL platform. The consensus tree of the ML and BI analyses was generated manually from the topology of the ML analysis, including bootstrap and posterior probability values, using the CorelDRAW editing package 2020.

Pairwise genetic distances among novel *Trichoderma* isolates and their closest known relatives were calculated from partial *tef1-α* sequence alignments. Sequences were aligned using MAFFT v.7 under default parameters, and p-distances were computed using the identity model implemented in BioPython (v.1.83). The resulting matrix was visualized as a hierarchically clustered heatmap (average linkage, Euclidean distances) using Seaborn (v.0.12). To identify fixed nucleotide differences supporting species delimitation, each novel taxon was compared against its closest relative. A position was considered diagnostic when all isolates of the new species shared a fixed nucleotide or indel state absent in the reference, excluding ambiguous bases (N). Diagnostic positions were highlighted by color-coded asterisks in alignments visualized using pymsaviz (v.0.4.2).

### 2.4 Morphological characterization

The macromorphological characterization was performed in PDA, CMD (2 g infusion corn meal, 20 g dextrose, 15 g agar), and SNA (1 g KH_2_PO_4_, 1 g KNO_3_, 0.5 g MgSO_4_, 0.5 g KCl, 0.2 g glucose, 0.2 g sucrose, 15 g agar) media. The strains grew for 7 days at 30 °C.

The microstructures, such as conidiophores, conidia, and chlamydospores, were analyzed using scanning electron microscopy (MEV) (JSM-IT500HR) at the Multiuser Center for the Analysis of Biomedical Phenomena at the Amazonas State University (CMABio). For this, the microculture was prepared on PDA at 30 °C for 7 days, fixed with Karnovsky, and then dehydrated in increasing concentrations of alcohol (30, 50, 70, 80, 90, and 100%) for 15 min each. The step with absolute alcohol was repeated twice. Drying was performed in a critical-point dryer (Leica EM CPD300), and samples were sputter-coated (DII-29010SCTR Smart Coater). Thirty measurements were taken for each structure (conidia, phialides, and chlamydospores) using an optical microscope with a Carl Zeiss Axio Imager v2 camera.

### 2.5 Dual culture assay

Discs with mycelia (5 mm) of the *Trichoderma* isolates and of the phytopathogens were inoculated in Petri dishes (90 mm) containing PDA culture medium in opposite directions at a distance of 50 mm from the edge. Dishes containing only the phytopathogen were used as a positive control. Inocula were incubated at 30 °C and evaluated after 21 days. Each assay was conducted in triplicate. The percentage of growth inhibition of phytopathogens was calculated according to the formula: Inhibition (%) = [(GC – GT)/GC] × 100, in which GC represents the mean diameter of the control, and GT represents the mean diameter of the treatment. The mycelial growth inhibition data were used to generate a grouped bar chart using the ggplot2 package in R software. Detailed information about the phytopathogens is plotted in Supplementary Table S1.

### 2.6 Genome sequencing, assembly, and quality assessment

Genome sequencing was performed by a third-party service using the Illumina NovaSeq 6,000 platform, generating 150-base-pair paired-end (150 bp PE) reads. Raw sequencing reads for all five isolates have been deposited in the NCBI Sequence Read Archive (SRA) under BioProject accession PRJNA1420911: TM14 (SRR37678991; BioSample SAMN55228064), TM26 (SRR37678990; SAMN55228065), TM42 (SRR37678989; SAMN55228066), TM58 (SRR37678988; SAMN55228067), and TM67 (SRR37678987; SAMN55228068).

Read quality was assessed using FastQC v0.11.9 to evaluate nucleotide quality distribution, adapter presence, and potential contaminants. Low-quality reads and adapter sequences were removed using Trimmomatic v0.39, applying a Phred quality score threshold of ≥30 and a minimum read length of 50 bp. Genome assembly was conducted using SPAdes v3.15.3, with default parameters for paired-end data. Assembly quality was evaluated using QUAST v5.2.0, considering statistics such as the number of contigs, N50, and total genome size.

Potential contamination was assessed using CheckM v1.2.2. Assembly statistics and annotation metrics for all sequenced isolates and reference genomes used in this work, including genome size, GC content, number of contigs, and N50/L50 values, are provided in Supplementary Table S2. Genome completeness was assessed using BUSCO (Benchmarking Universal Single-Copy Orthologs) against the appropriate lineage dataset, and completeness scores and genome accession numbers are reported in Supplementary Table S3.

### 2.7 CAZyme analysis

The complete genome was subjected to protein prediction using Augustus on the Galaxy platform^3^, with *Fusarium graminearum* used as the training set. Subsequently, the predicted proteins were analyzed using the dbCAN3 web server for automated carbohydrate-active enzyme annotation^4^. Annotations were considered reliable when supported by at least two of the three prediction tools (HMMER, DIAMOND, and eCAMI). The resulting annotations were processed using a custom Python script that employed the pandas library to count the occurrence of each CAZyme family across all annotated proteins. Families were then classified according to their primary substrate preference (bacterial cell wall, cellulose, fungal cell wall, hemicellulose, lignin, and pectin) based on functional annotations from the CAZy database. A heatmap was generated using the *pheatmap* package in R to visualize protein count distributions across isolates and substrate categories.

### 2.8 Genome mining

Genome mining for biosynthetic gene clusters (BGCs) was performed using FungiSMASH^5^ to predict and annotate secondary metabolite biosynthetic pathways. Detection strictness was set to relaxed, enabling identification of both well-defined clusters containing all required parts and partial clusters missing one or more functional parts. All extra features were enabled, including KnownClusterBlast, ClusterBlast, SubClusterBlast, MIBiG cluster comparison, ActiveSiteFinder, RREFinder, Cluster Pfam analysis, Pfam-based GO term annotation, TIGRFam analysis, and NCBI genomic context links. The identified BGCs were classified based on homology to known clusters and their functional annotations.

Clusters showing similarity to BGCs deposited in the MIBiG repository were selected for synteny analysis using clinker and clustermap.js (Gilchrist and Chooi, 2021), which aligns and visualizes gene arrangements within and across clusters. Comparative visualizations of BGC architectures across genomes were generated to identify conserved regions and structural variations, supporting the assessment of biosynthetic potential for specific compound classes. Figures were manually edited using CorelDRAW 2020.

## 3 Results

### 3.1 Phylogenetic analysis and species delimitation

Phylogenetic analysis of 44 isolates utilizing tef1-*α* sequences unveiled that 36 were assigned to nine known *Trichoderma* species: *T. lentiforme* (19), *T. afroharzianum* (5), *T. rifaii* (1), *T. endophyticum* (1), *T. cyanodichotomus* (5), *T. parareesei* (1), *T. ovalisporum* (1), *T. awajun* (1), and *T. capillare* (2). Furthermore, eight isolates clustered into five distinct clades, suggesting the occurrence of five novel species with high bootstrap support and concordant with species delimitation thresholds (*rpb2* ≥ 99% and *tef1-α* ≥ 97%) (Supplementary Figure 1).

**Figure 2:**
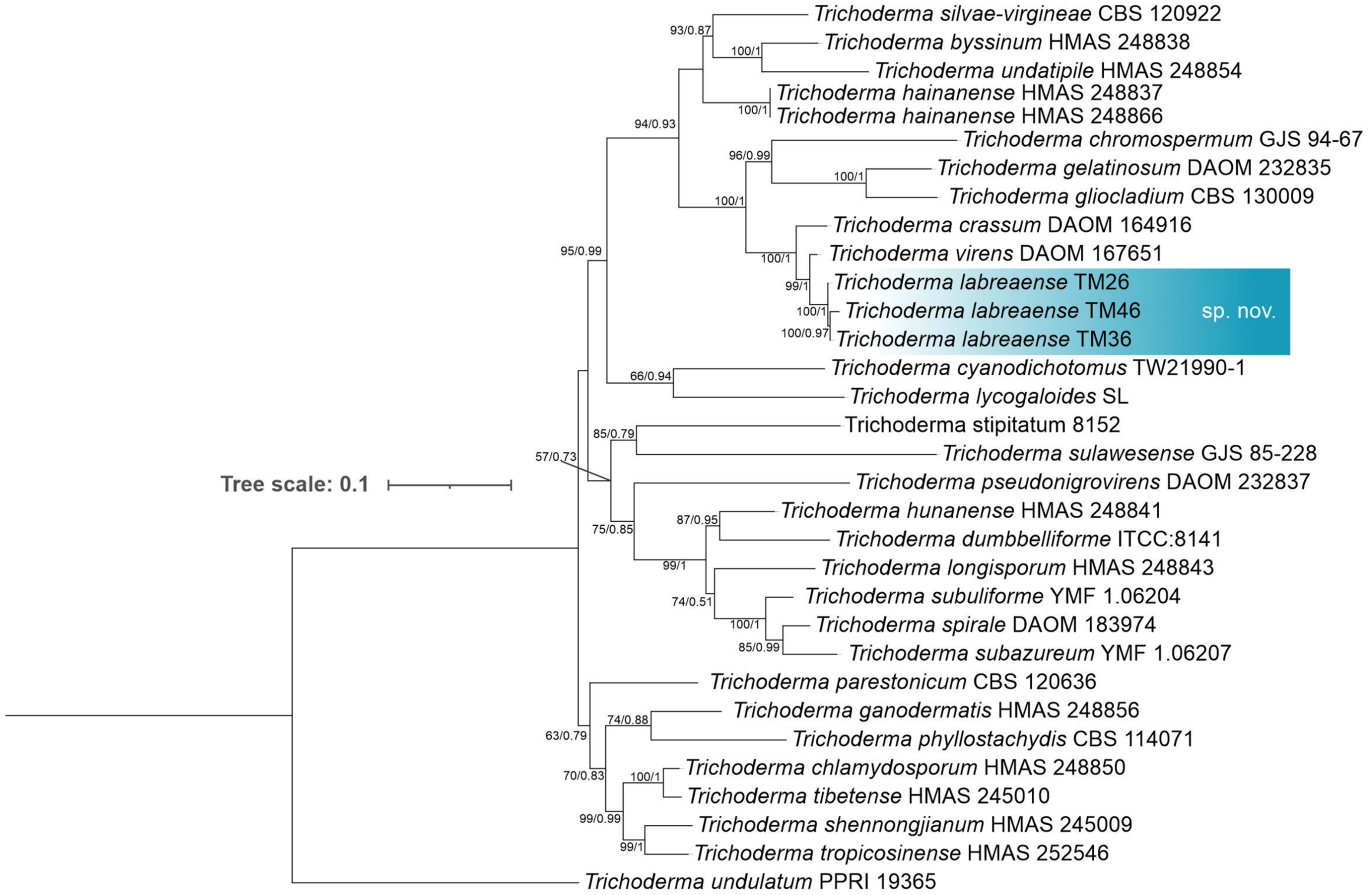
Phylogram constructed based on the alignment of *tef1-α* and *rpb2* loci of *Trichoderma* species closely related to *Trichoderma labreaense*. The isolates obtained in this study are marked in blue. The numbers on the branches indicate the support for each branch (ML/PP).

**Figure 3:**
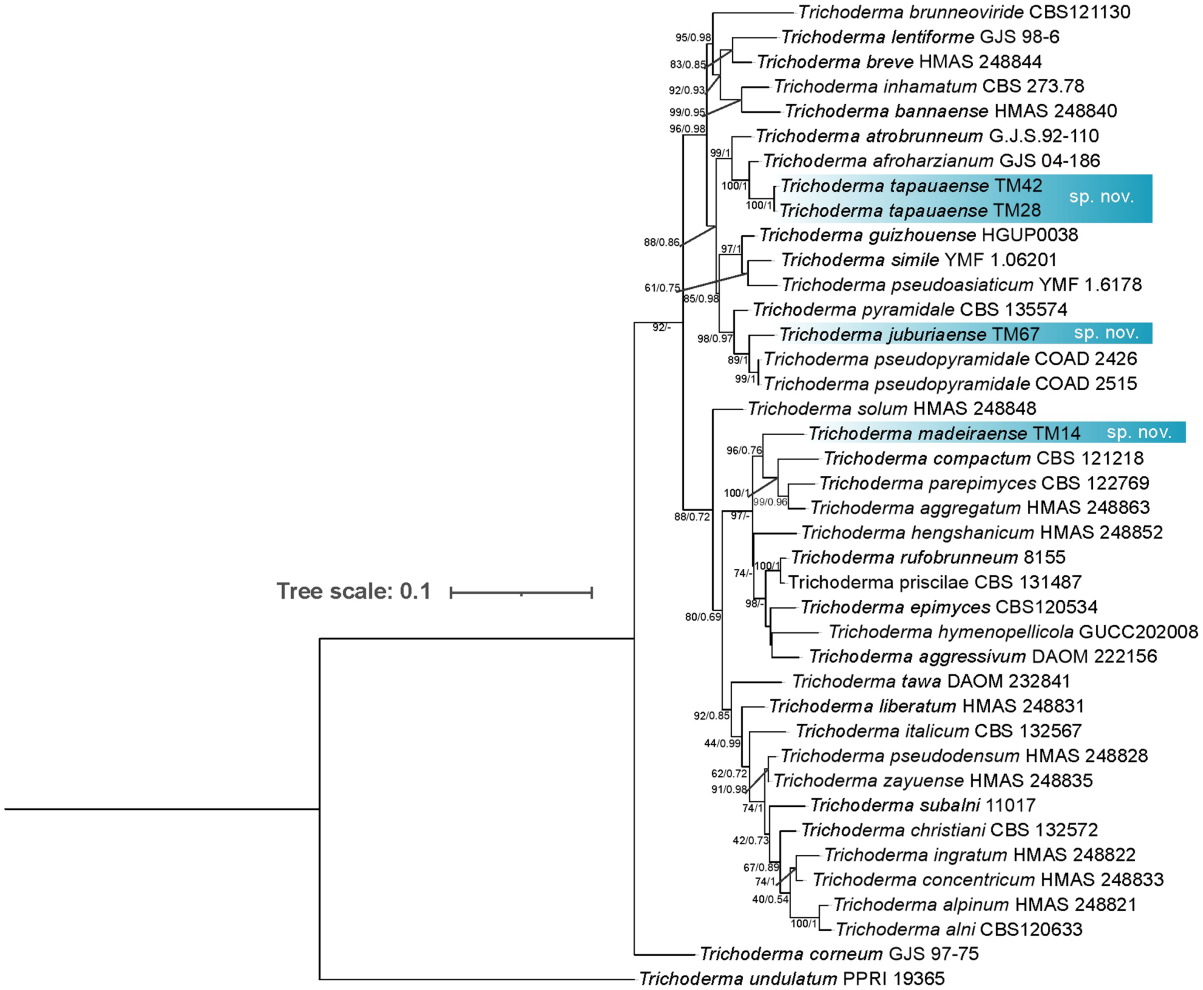
Phylogram constructed based on the alignment of *tef1-α* and *rpb2* loci of *Trichoderma* species closely related to *Trichoderma tapauaense*, *Trichoderma juburiaense*, and *Trichoderma madeiraense*. The isolates obtained in this study are marked in blue. The numbers on the branches indicate the support for the branches (ML/PP).

**Figure 4:**
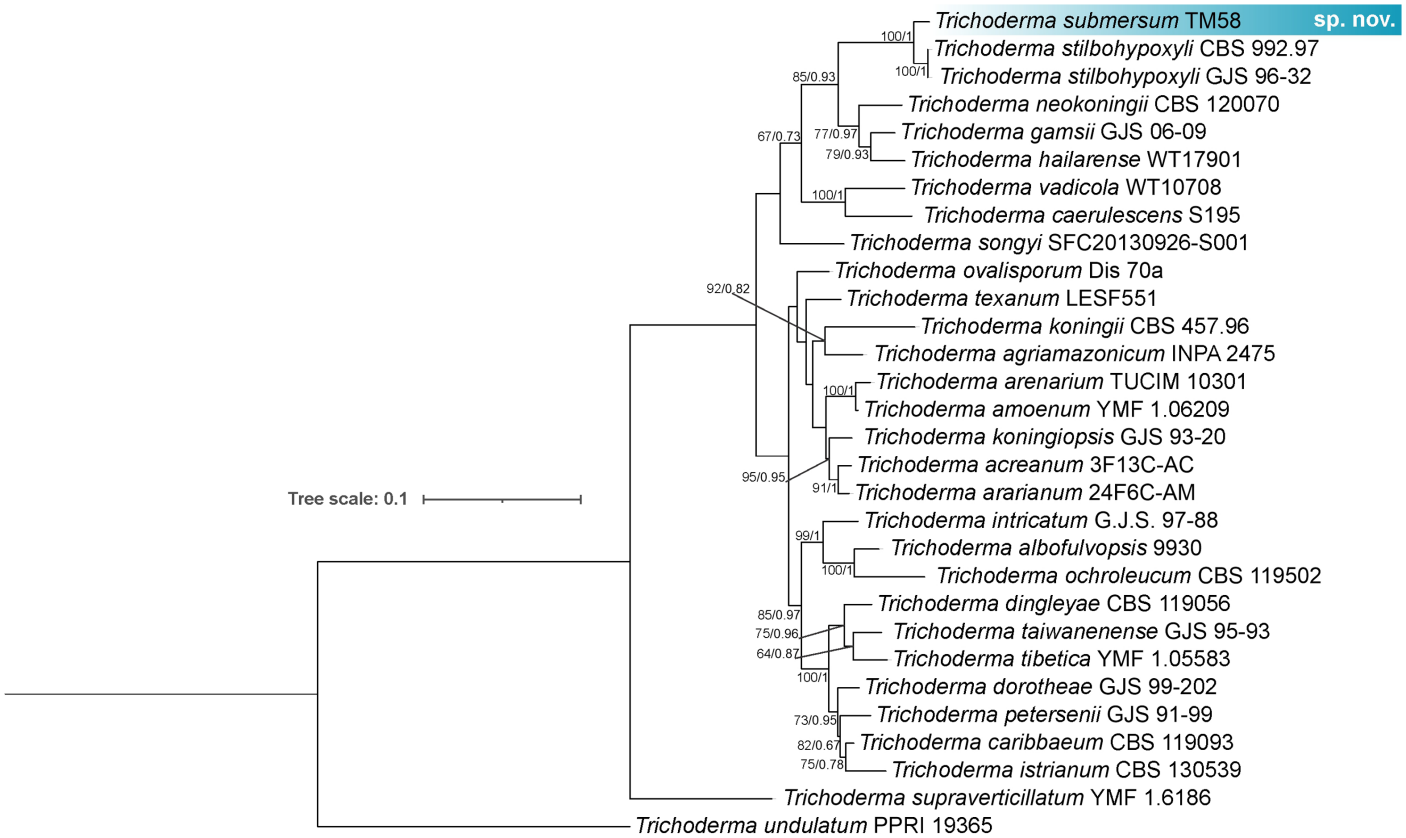
Phylogram constructed based on the alignment of *tef1-α* and *rpb2* loci of *Trichoderma* species closely related to *Trichoderma submersum*. The isolates obtained in this study are marked in blue. The numbers in the branches indicating the branches support (ML/PP).

Phylogenetic analysis based on concatenated *tef1-α* and *rpb2* sequences revealed five distinct lineages corresponding to new species within the genus *Trichoderma*. Isolates TM26, TM36, and TM46 formed a clade closely related to *T. virens*, with TM26 designated as the type strain. This lineage exhibits 97% sequence similarity with *T. virens* in *tef1-α* (p-distance = 0.029) and 98.01% in *rpb2*, and is supported by 16 diagnostic nucleotide positions relative to *T. virens* DAOM 167651. This lineage is consequently described here as *Trichoderma labreaense* (Figure 2). Isolates TM28 and TM42 clustered with *T. afroharzianum*, showing 95.26% similarity in *tef1-α* (p-distance = 0.039) and 99.06% in *rpb2*, and are distinguished by 22 diagnostic positions relative to *T. afroharzianum* GJS 04–186, including a diagnostic 6 bp deletion at positions 71–76 of the *tef1-α* alignment. These isolates were designated as *Trichoderma tapauaense* (Figure 3). Isolate TM67 grouped closely with *T. pseudopyramidale*, displaying 95.44% similarity in *tef1-α* (p-distance = 0.061) and 99% in *rpb2*, supported by 34 diagnostic positions, and was named *Trichoderma juburiaense* (Figure 3). Isolate TM58 formed a clade with *T. stilbohypoxyli*, exhibiting 96.84% similarity in *tef1-α* (p-distance = 0.031) and 17 diagnostic nucleotide positions. Since *rpb2* sequence data for the *T. stilbohypoxyli* type strain are not available, phylogenetic placement was based solely on *tef1-α*, and this isolate was designated as *Trichoderma submersum* (Figure 4). Isolate TM14 clustered with *T. compactum*, *T. aggregatum*, and *T. parepimyces*, showing approximately 97% similarity in both *tef1-α* and *rpb2* (p-distance = 0.165 relative to *T. compactum* CBS 121218), supported by 50 diagnostic positions, and was described as *Trichoderma madeiraense* (Figure 3). Pairwise genetic distances among all novel species and their closest relatives are summarized in Supplementary Figure 2, and diagnostic nucleotide positions for each lineage are illustrated in Supplementary Figure 3.

### 3.2 Taxonomy

*Trichoderma madeiraense* (TM14) T. F. Sousa, R. Gwinner, G. S. Castro, I. J. S. Silva & G. F. Silva, sp. nov.

Mycobank: MB860010.

Etymology: refers to the river from which the type was isolated.

*Typification:* BRAZIL: AMAZONAS. ITACOATIARA, in the Fazendinha riverside Amazon community, 07°03′12.7 S 62°51′00.9 W, 1 m depth, from sediments of Madeira River, 01 October 2018, collected by Jeferson Chagas da Cruz, isolated by Raoni Gwiner, URM 9358 (ex-type culture). GenBank accessions: ITS = PV911309, *tef1-α* = PV695515, *rpb2* = PX757594.

**Figure 5:**
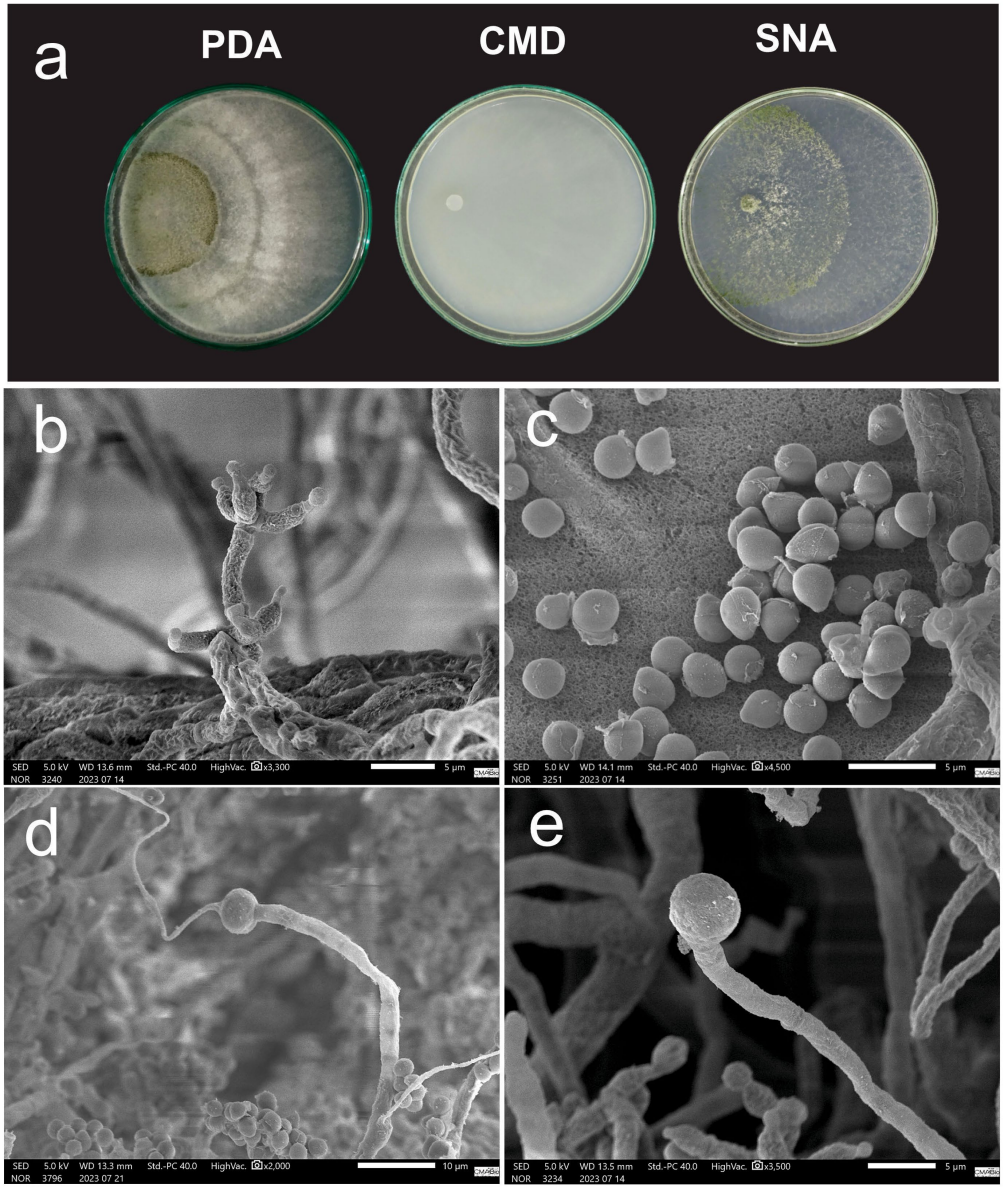
*Trichoderma madeiraense* cultivated in PDA, CMD, and SNA medium for 7 days: **(a)** Conidiophore; **(b)** Conidia; **(c)** Intercalary chlamydospores; **(d)** Terminal chlamydospores.

*Macromorphology*: Colonies grown on PDA at 30 °C for 7 days exhibit white cottony mycelium with concentric rings formed by sparse mycelium and high conidiation in the center of the culture. Colonies on CMD grown at 30 °C for 7 days exhibit sparse mycelium growth with low conidiation and absence of pustule formation. Colonies grown on CMD at 30°C for 7 days showed sparse mycelium with low conidiation and no pustule formation (Figure 5a).

*Micromorphology*: Conidiophores present smooth walls containing 3–4 ampulliform to lageniform phialides measuring 3.98–4.48 μm (*M* = 4.11 μm) × 1.56–1.88 μm (*M* = 1.69 μm) with the presence of greenish to brown, globose or sub-globose conidia measuring 2.44–3.13 μm (*M* = 2.66 μm) × 2.18–2.48 μm (*M* = 2.32 μm) (Figures 5b,c). Presence of terminal globose chlamydospores with 4.79–6.42 μm (*M* = 5.45 μm) × 4.64–6.57 μm (*M* = 5.23 μm) (Figures 5d,e).

*Notes*: Differs from *T. compactum* by the smallest length of phialides and conidia structures.

*Trichoderma labreaense* (TM26) T. F. Sousa, R. Gwinner, G. S. Castro, I. J. S. Silva & G. F. Silva, sp. nov.

Mycobank: MB860011.

*Etymology:* Refers to the location type.

*Typification:* BRASIL: AMAZONAS. LÁBREA, S 07°28′22.9 W 065°18′10.1, 0.3 m depth, from sediments of Purus River, 27 November 2018, collected by Jeferson Chagas da Cruz and isolated by Raoni Gwiner, URM 9354 (ex-type culture). GenBank accessions: ITS=PV911310, *tef1-α =* PV695519, *rpb2 =* PX757597.

**Figure 6:**
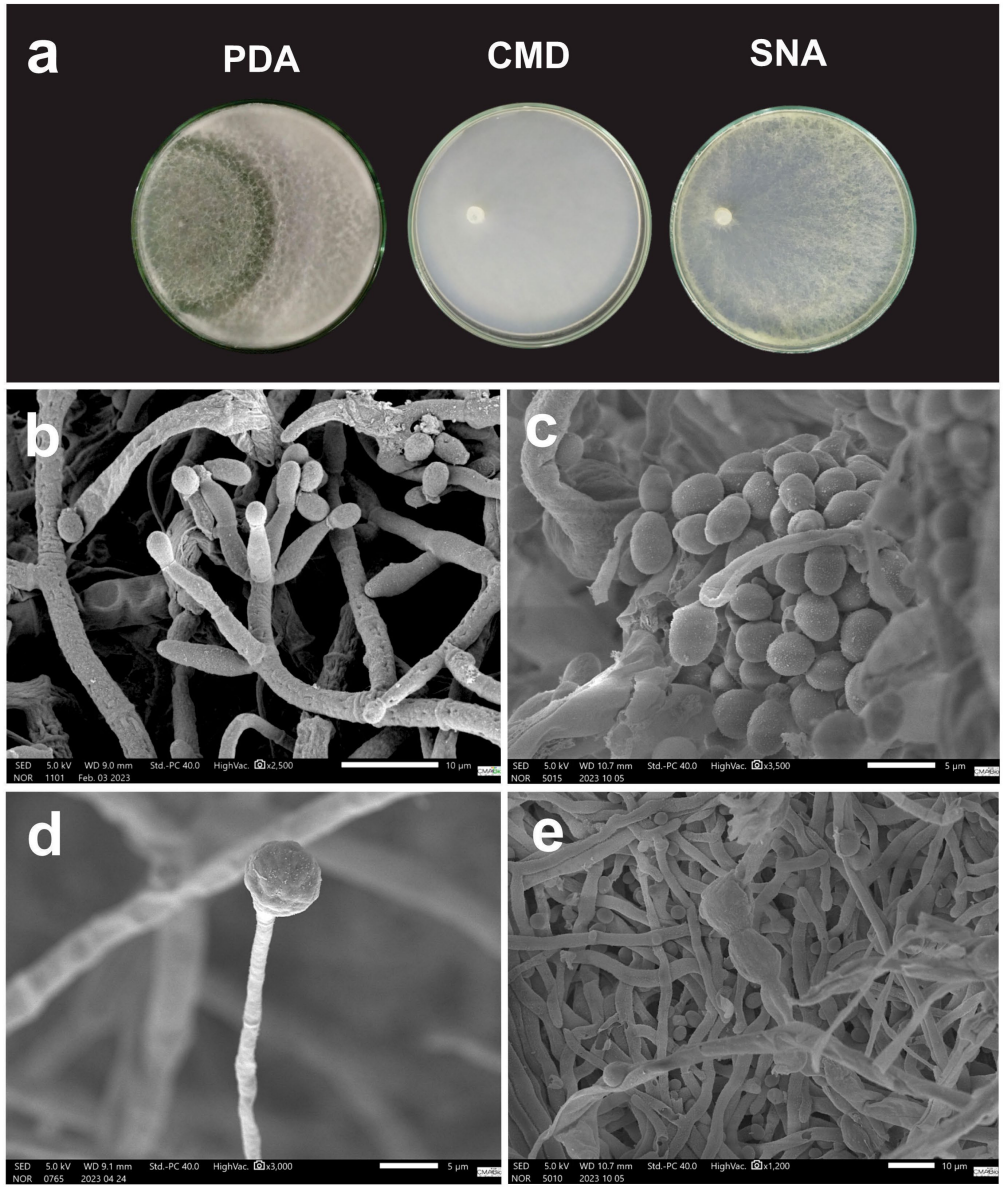
**(a)** *Trichoderma labreaense* cultivated in PDA, CMD and SNA medium for 7 days. **(b)** Conidiophore. **(c)** Conidia. **(d,e)** Chlamydospores.

*Macromorphology:* Colonies grown on PDA at 30 °C for 7 days exhibit white cottony mycelial and high conidiation in the center of the culture. Colonies grown on CMD at 30°C for 7 days displayed aerial mycelium with low conidiation, absence of pustule formation, and yellow pigment diffusing into the culture medium. (Figure 6a).

*Micromorphology:* Conidiophores present smooth walls containing 2–4 lageniform phialides measuring 7.80–22.01 μm (*M* = 12.27 μm) × 1.80–3.32 μm (*M* = 2.61 μm) with the presence of green, globose to ovoid conidia measuring 3.59–5.00 μm (*M* = 4.32 μm) × 3.15–4.60 μm (*M* = 3.69 μm) (Figures 6b,c). Presence of terminal globose chlamydospores with 9.21–15.00 μm (*M* = 11.55 μm) × 8.73–13.57 μm (*M* = 10.99 μm) (Figures 6d,e).

*Sexual stage*: Not observed.

*Distribution*: Brazil, Amazonas.

*Notes*: It differs from *Trichoderma virens* by having a greater phialide length.

*Trichoderma tapauaense* (TM42) T. F. Sousa, R. Gwinner, G. S. Castro, I. J. S. Silva & G. F. Silva, sp. nov.

Mycobank: MB860012.

*Etymology:* Refers to the location type.

*Typification:* BRAZIL: AMAZONAS. TAPAUÁ, S 05°24′56.2 W 063°04′55.1, 4 m depth, from sediments of Purus River, 05 December 2018, collected by Jeferson Chagas da Cruz and isolated by Raoni Gwiner, URM 9355 (ex-type culture). GenBank accessions: ITS = PV911311 *tef1-α =* PV695530, *rpb2* = PX757595.

**Figure 7:**
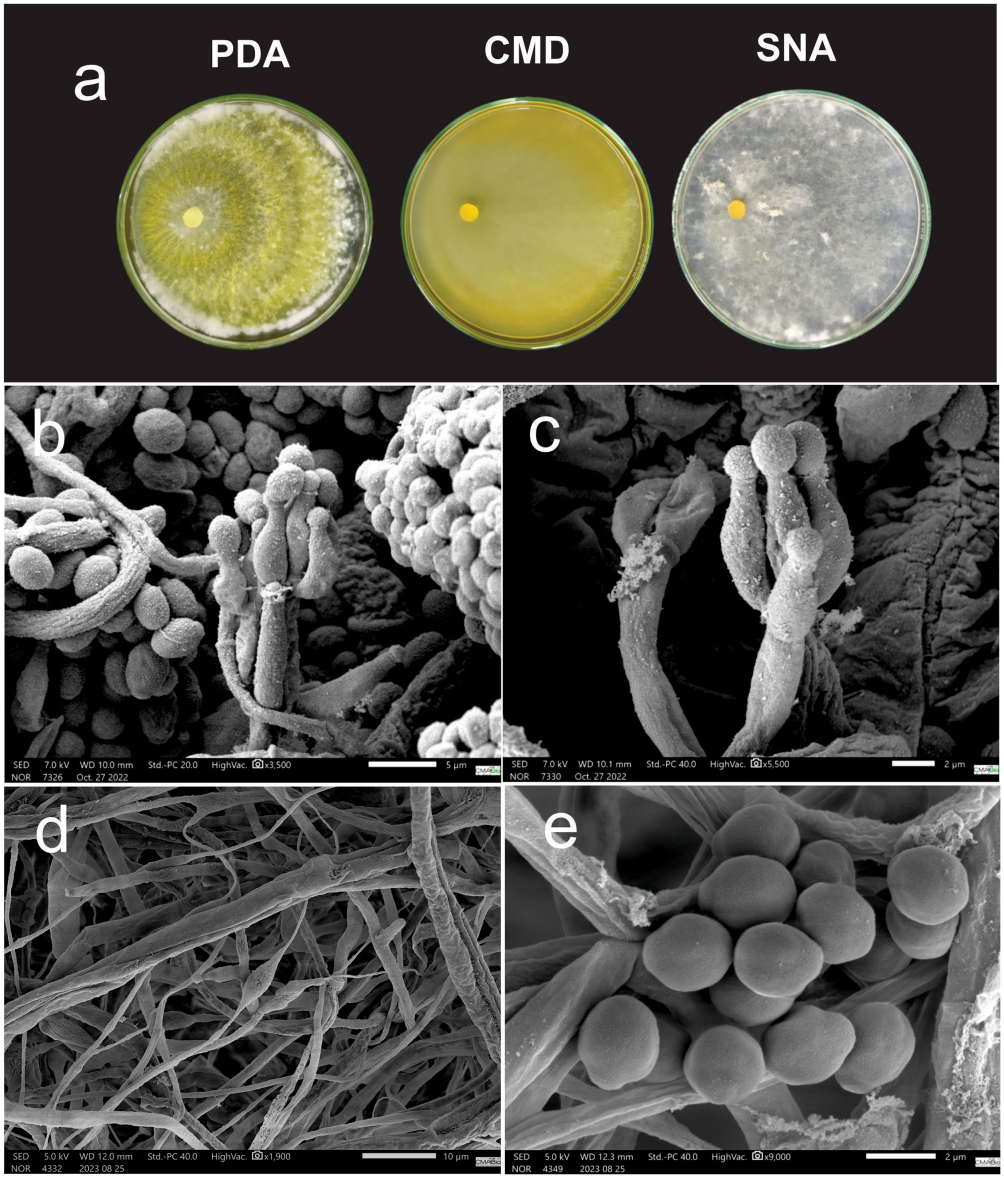
**(a)** *Trichoderma tapauaense* cultivated in PDA, CMD and SNA medium for 7 days. **(b,c)** Conidiophore. **(d)** Intercalary chlamydospores. **(e)** Conidia.

*Macromorphology:* Colonies grown on PDA at 30 °C for 7 days display abundant yellow aerial mycelia with pustule formations and concentric rings. Colonies on CMD grown at 30 °C for 7 days exhibit sparse yellow mycelium with low conidiation, the absence of pustule formations, and high levels of yellow extrolites diffusing in the culture media. Colonies in SNA growing at 30 °C for 7 days present a sparse mycelium with low conidiation and absence of pigmentation (Figure 7a).

*Micromorphology:* Conidiophores present smooth walls containing 2–4 lageniform to ampulliform phialides measuring 5.56–13.02 μm (*M* = 8.80 μm) × 1.57–3.73 μm (*M* = 2.28 μm) with the presence of yellow-green, sub-globose to ovoid conidia measuring 2.96–4.63 μm (M = 3.92 μm) × 2.51–3.95 μm (M = 3.27 μm) (Figures 7b,c,e). Intercalary chlamydospores were observed (Figure 7d) Terminal chlamydospores not observed.

*Sexual stage*: Not observed.

*Distribution*: Brazil, Amazonas.

*Notes*: differs from *Trichoderma afroharzianum* by frequent yellow pigmentation.

*Trichoderma submersum* (TM58) T. F. Sousa, R. Gwinner, G. S. Castro, I. J. S. Silva & G. F. Silva, sp. nov.

Mycobank: MB860013.

*Etymology:* Referring to the submerged sediment sample where the fungus was isolated.

*Typification:* BRAZIL: AMAZONAS. JURUÁ, S 03°41′36.4 W 066°11′33.9, 5.5 m depth, from sediments of the Juruá River, 01 January 2019, collected by Thiago Fernandes Sousa and isolated by Raoni Gwiner, URM 9356 (ex-type culture). GenBank accessions: ITS=PV911312, *tef1-α =* PV695542, *rpb2* = PX757599.

**Figure 8:**
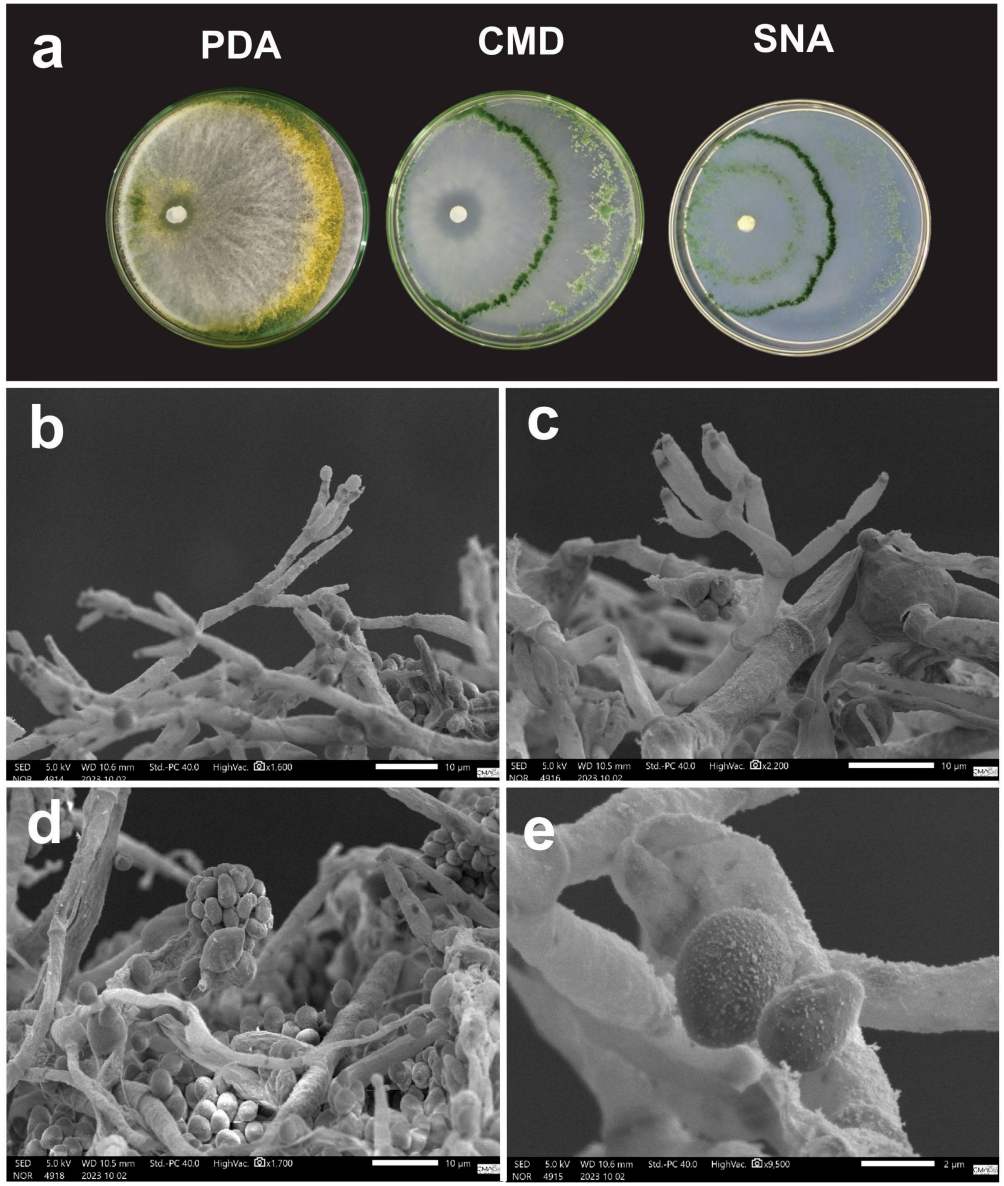
**(a)** *Trichoderma submersum* cultivated in PDA, CMD and SNA medium for 7 days. **(b,c)** Conidiophore. **(d)** Intercalary chlamydospore and conidia. **(e)** Conidia.

*Macromorphology:* Colonies grown on PDA at 30 °C for 7 days present abundant aerial mycelia and high production of green and yellow conidia in the border. Colonies on CMD grown at 30 °C for 7 days show sparse mycelia with high conidiation, concentric rings, and pustule formation mainly along the borders and within concentric rings; pigment diffusing in the culture media was not observed. Colonies in SNA grown at 30 °C for 7 days present a sparse mycelium with well-defined concentric rings, high conidiation and pustule formation along the border, and no pigment diffusion in the culture medium (Figure 8a).

*Micromorphology:* Conidiophores are verticillium-like and contain 6–7 ampulliform phialides measuring 7.06–9.83 μm (M = 8.75 μm) × 1.75–2.92 μm (M = 2.13 μm) with the presence of yellow-green, ovoid conidia measuring 3.57–4.75 μm (M = 4.16 μm) × 2.61–3.47 μm (M = 2.95 μm) (Figures 8b,c,e). Intercalary chlamydospores were also observed (Figure 8d). Terminal chlamydospores not observed.

*Notes*: differs from *Trichoderma stilbohypoxyli* by having greater conidia and phialides.

*Trichoderma juburiaense* (TM67) T. F. Sousa, R. Gwinner, I. J. S. Silva, G. S. Castro & G. F. Silva, sp. nov.

Mycobank: MB860014.

*Etymology:* Refers to the location type.

*Typification:* BRAZIL: AMAZONAS. CARAUARI, S 05°11′20.4 W 067°13′41.8, in the Juburiá riverside Amazon community, 2 m depth, from sediments of Juruá River, 23 January 2019, collected by Thiago Fernandes Sousa and isolated by Raoni Gwiner, URM 9357 (ex-type culture). GenBank accessions: ITS = PV911313, *tef1-α =* PV695547, *rpb2* = PX757596.

**Figure 9:**
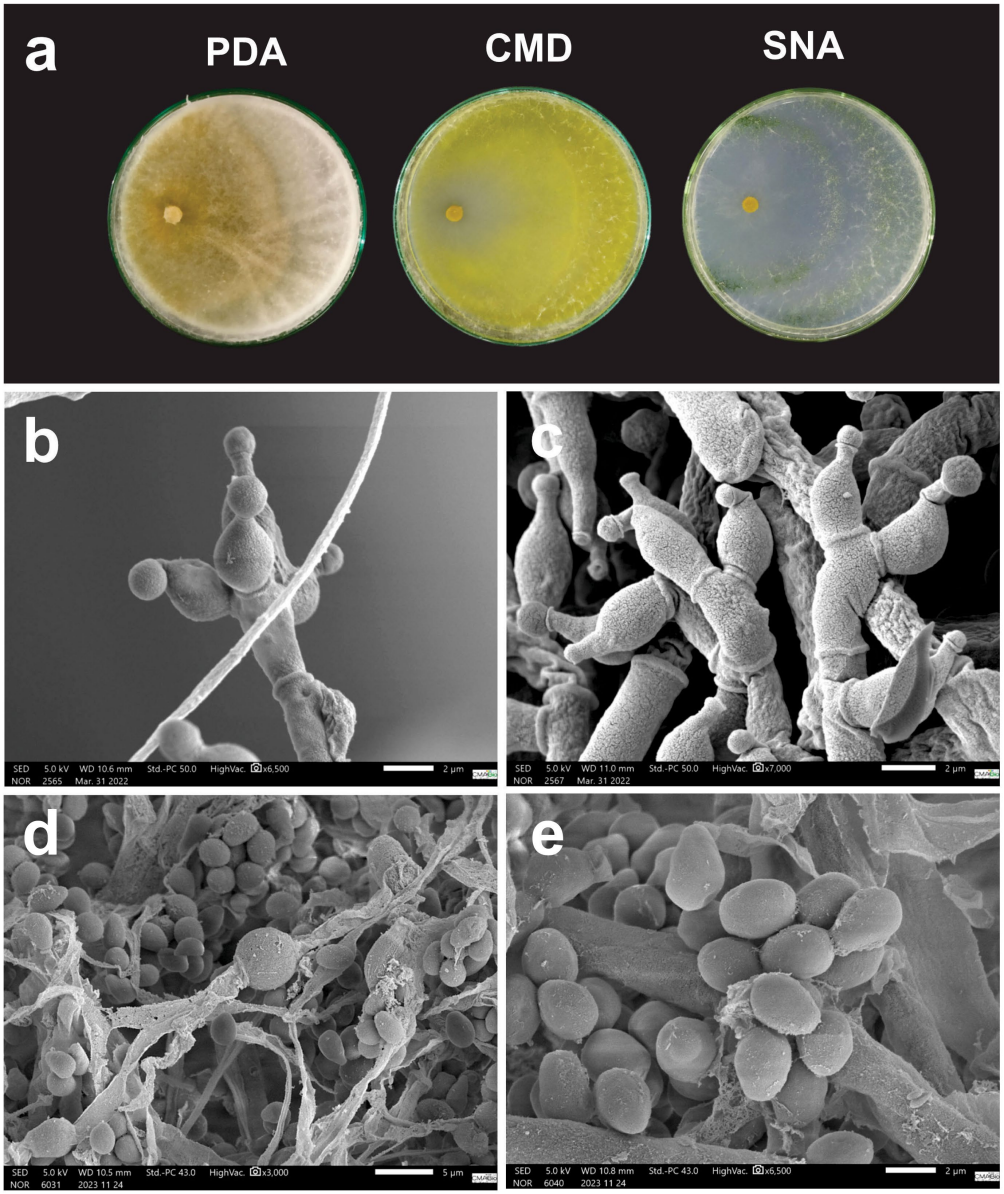
**(a)** *Trichoderma juburiaense* cultivated in PDA, CMD, and SNA medium for 7 days. **(b,c)** Conidiophore. **(d)** Intercalary chlamydospores. **(e)** Conidia.

*Macromorphology:* Colonies grown on PDA at 30 °C for 7 days present abundant aerial mycelia with orange-to-red pigmentation and low conidiation. Colonies on CMD grown at 30 °C for 7 days present aerial mycelia with low conidiation, an absence of pustule formation, and yellow pigment diffusing in the culture media, which was not observed. Colonies in SNA grown at 30 °C for 7 days exhibit a sparse mycelium with well-defined concentric rings, high conidiation and pustule formation along the border, and the absence of pigment diffusion in the culture media (Figure 9a).

*Micromorphology:* Conidiophores present smooth walls containing 2–4 ampulliform, broadly ellipsoid small phialides measuring 4.58–8.50 μm (*M* = 6.45 μm) × 1.41–2.69 μm (*M* = 1.98 μm) with the presence of green, sub-globose to ovoid conidia measuring 2.64–4.29 μm (*M* = 3.46 μm) × 2.00–3.02 μm (*M* = 2.55 μm) (Figures 9b,c,e). Intercalary chlamydospores were observed (Figure 9d). Presence of terminal globose chlamydospores with 3.86–8.66 μm (*M* = 5.59 μm) × 2.74–6.12 μm (*M* = 4.46 μm).

*Sexual stage*: Not observed.

*Distribution*: Brazil, Amazonas.

*Notes*: differs from *Trichoderma pseudopyramidale* by having greater conidia and yellow to orange pigment diffusing in culture media.

### 3.3 Antagonistic activity of new *Trichoderma* species against different phytopathogen species

**Figure 10:**
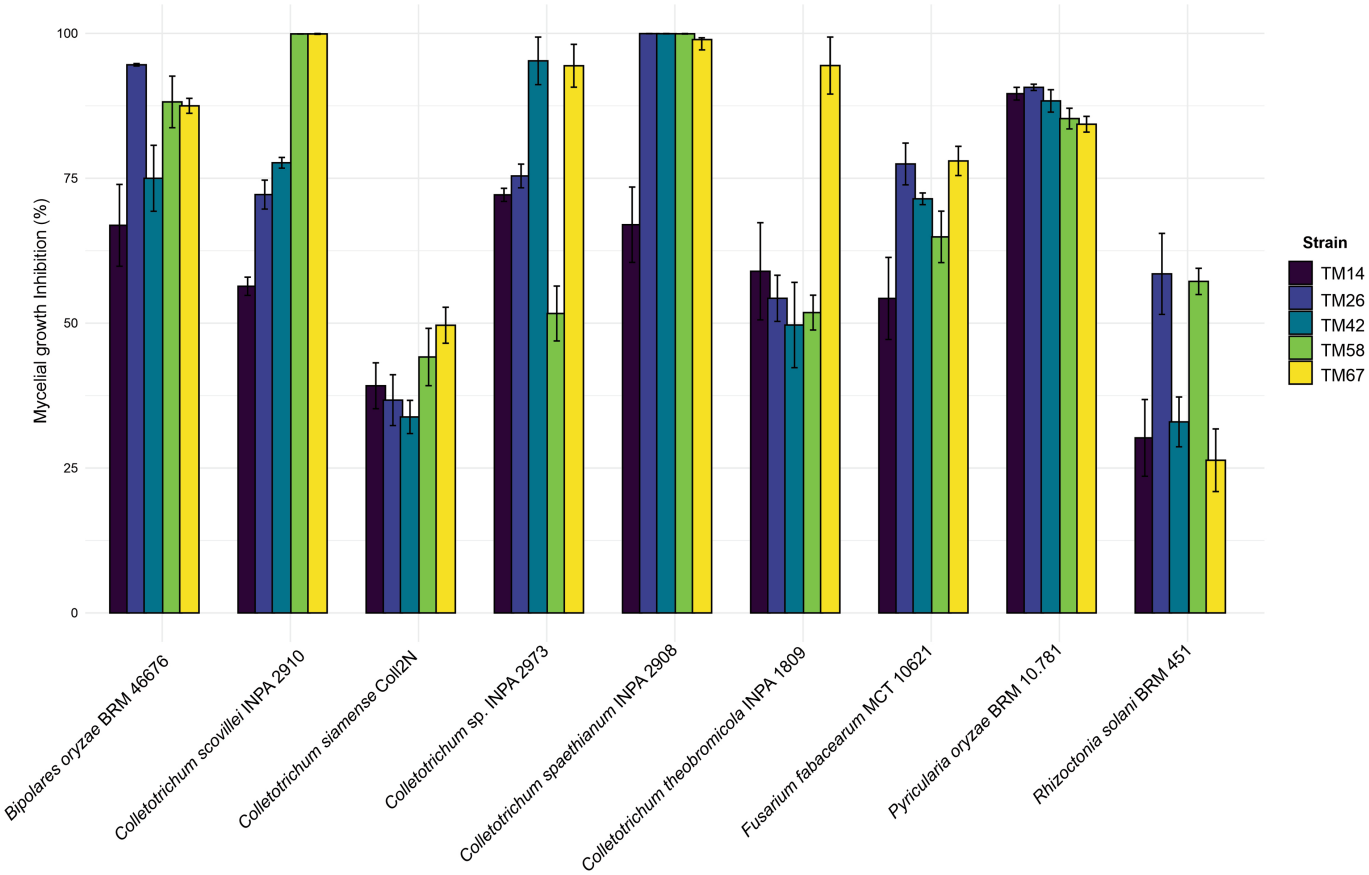
*In vitro* antagonistic activity of a novel *Trichoderma* species against nine different phytopathogens that infect crops of agricultural importance. The bars represent the mean inhibition percentage, and the error bars show the standard deviation. Each color corresponds to a different *Trichoderma* strain.

The new species exhibited biocontrol activity against all tested phytopathogens, with efficacy varying by target organism. Against *Colletotrichum spaethianum* INPA 2908, nearly all isolates achieved mycelial growth inhibition above 98% after 21 days of incubation, with rates ranging from 66.98% (TM14) to 99.96% (TM26). The inhibitory spectrum varied considerably among other *Colletotrichum* species tested. For *C. theobromicola* INPA 1809, only TM67 exceeded 94% inhibition, while all other isolates remained below 59%, with TM42 showing the lowest activity at 49.67%. *C. siamense* Coll2N exhibited inhibition levels ranging from 33.81 to 49.64%, with TM67 and TM42 showing the highest and lowest values, respectively. Against *C. scovillei* INPA 2910, the isolates TM48 and TM67 achieved inhibition rates above 95%, with TM58 and TM67 reaching 99.61%, while TM14 showed 49.64%. For *Colletotrichum* sp. INPA 2973, TM42, and TM67 exhibited MGI values above 94%, whereas the remaining isolates showed inhibition below 75%, with TM58 at 51.66% (Figure 10).

The *Trichoderma* isolates also showed strong inhibitory effects against rice pathogens. For *Pyricularia oryzae* BRM 10.781, all isolates exceeded 84% inhibition, with TM26 achieving 90.69–91.44% and TM67 showing 84.32%. Against *Bipolaris oryzae* BRM 46676, inhibition ranged from 66.88% (TM14) to 94.58% (TM26), with other isolates exceeding 74.99%. For *Fusarium fabacearum* MCT 10621, inhibition ranged from 54.26 to 77.46%, with TM67 and TM14 showing the highest and lowest values, respectively. Against *Rhizoctonia solani* BRM 45111, inhibition varied from 26.34 to 68.05%, with TM26 and TM58 exceeding 58%, while TM67 showed the lowest inhibition at 26.34% (Figure 10).

### 3.4 Genome assembly and quality assessment

The genomes of the five novel *Trichoderma* species sequenced in this study ranged from 38.1 Mb (*T. submersum* TM58) to 43.4 Mb (*T. tapauaense* TM42), with GC content varying between 43.2 and 48.9% (Supplementary Table S3). Assembly contiguity varied among isolates, with N50 values ranging from 274 kb (TM42) to 1,865 kb (TM26), reflecting differences in sequencing depth and assembly quality. BUSCO completeness assessment against the hypocreales_odb10 dataset revealed high genome completeness across all assemblies, with complete BUSCO scores ranging from 98.9 to 99.1%, comparable to the reference genomes used in this study (98.7–99.0%) (Supplementary Table S2). These results indicate that the genome assemblies are of sufficient quality to support reliable downstream functional annotation, including CAZyme profiling and biosynthetic gene cluster prediction.

### 3.5 CAZyme profile

**Figure 11:**
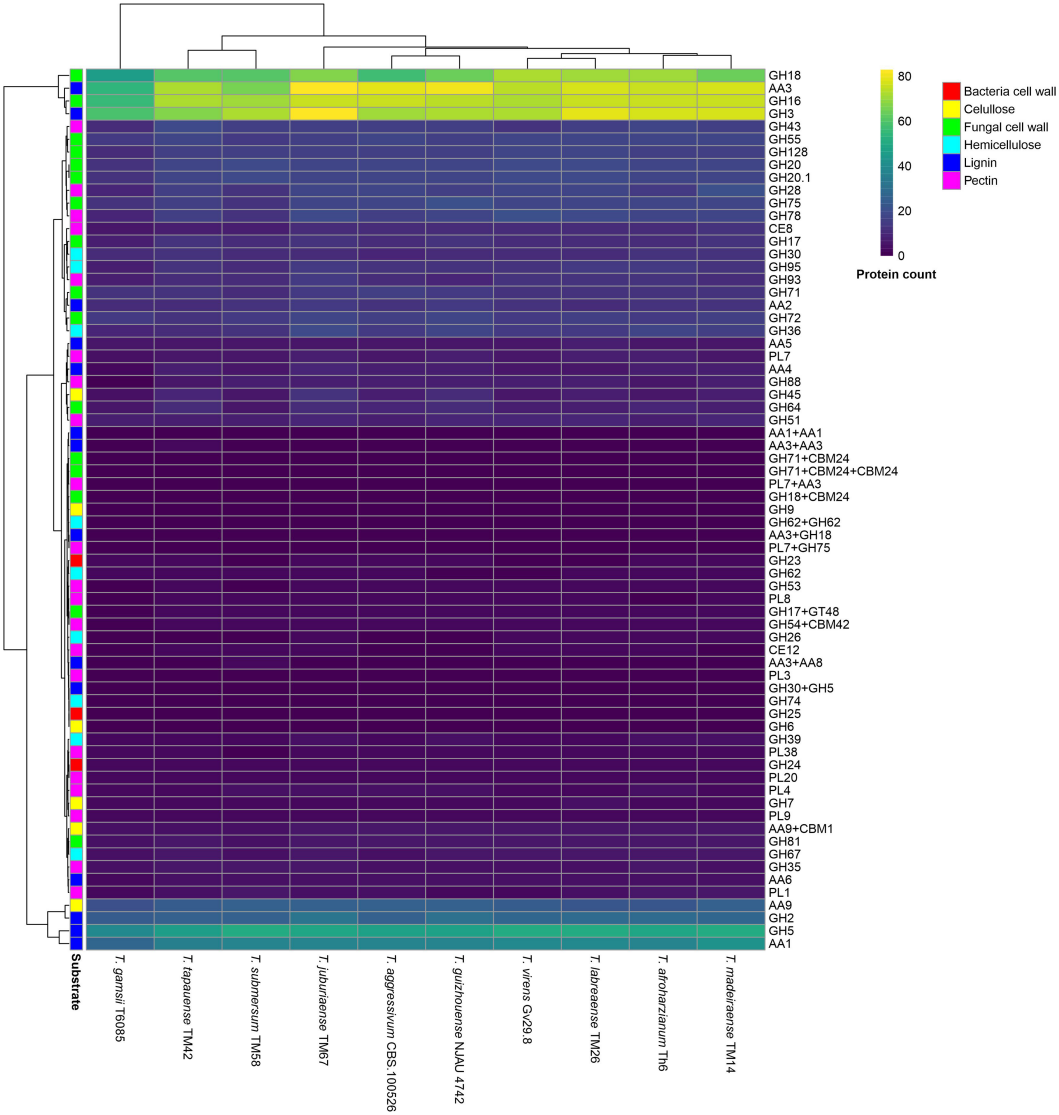
CAZyme heatmap is sorted by substrate preference, based on 11 different isolates from five newly described species in this study and five closely related species. GH, glycosyl hydrolases; AA, auxiliary activity; CE, carbohydrate esterase; PL, polysaccharide lyase.

All analyzed isolates showed a high abundance of glycoside hydrolases (GH) associated with fungal cell wall degradation, especially from families GH16, GH18, and GH72. Notably, *T. labreaense* sp. nov. TM26 exhibited the highest number of GH18 annotations (75), while *T. juburiaense* sp. nov. TM67 had the highest count for GH16 (76), and *T. madeiraense* sp. nov. TM14 recorded the highest for GH72 (Figure 11).

The enzymes involved in bacterial cell wall hydrolysis were detected in lower abundance (lysozymes) and highly conserved, with highlights for families GH23, GH24, and GH25. The GH24 family maintained uniform values among the isolates, whereas GH25 was not annotated in TM26 (Figure 11).

For pectin hydrolysis, *T. madeiraense* sp. nov. TM14 and *T. virens* Gv29-8 exhibited the highest values (20 and 18, respectively), indicating potential alternatives in breaking down this substrate. Conversely, the *T. gamsii* strain T6085 and *T. submersum* sp. nov. TM58 had the fewest proteins in this family (9 and 13, respectively). Furthermore, PL7 (polysaccharide lyase) was well represented, with *T. tapauaense* sp. nov. TM42 and *T. juburiaense* sp. nov. TM67 showing the highest values for PL7 (8) (Figure 11).

Cellulose hydrolysis was primarily driven by enzymes from the GH3, GH45, AA9, and AA3 families. Notably, *T. tapauaense* TM42 and *T. guizhouense* strain NJAU 4742 exhibited the highest number of GH45 annotations, with 9 and 12 proteins, respectively. The AA9 family was highly represented across all isolates, with values ranging from 23 to 25, highlighting its conserved role in cellulose hydrolysis. For lignin hydrolysis, a notable expansion of the GH3 and AA3 families was observed, particularly in TM26 (79 and 77 proteins, respectively). Furthermore, TM42 was the only isolate in which more than one protein contained the AA3 + AA3 domain composition (Figure 11).

The hydrolysis of hemicellulose in the analyzed *Trichoderma* species was mainly supported by enzymes from the GH30, GH36, and GH95 families. The GH36 family exhibited the highest variability, with TM67 presenting the highest number of annotations (19), while other isolates ranged from 9 to 18. The GH30 family was consistently abundant across all isolates, with values between 9 and 12, suggesting a conserved role in hemicellulose hydrolysis. Similarly, GH95 was well represented, with TM26 showing the highest count (14), followed closely by other isolates, ranging from 8 to 14 (Figure 11).

Among the less-represented families for hemicellulose hydrolysis, GH39 exhibited the lowest counts across all species, with TM14 showing the highest count (4). The GH26 family was detected in a few isolates, including TM14, TM26, and TM67, with up to 2 annotations, while it was absent in others. Furthermore, GH62 was relatively conserved across isolates, typically with 2 annotations, except for *T. guizhouense* NJAU 4742, which had a lower count (1). A GH62 + GH62 duplication was exclusively identified in *T. virens* Gv29-8. Lastly, GH74, an enzyme involved in xyloglucan hydrolysis, was poorly annotated, with only one annotation in most isolates, emphasizing its limited expansion in these *Trichoderma* species (Figure 11).

### 3.6 Genome mining

**Figure 12:**
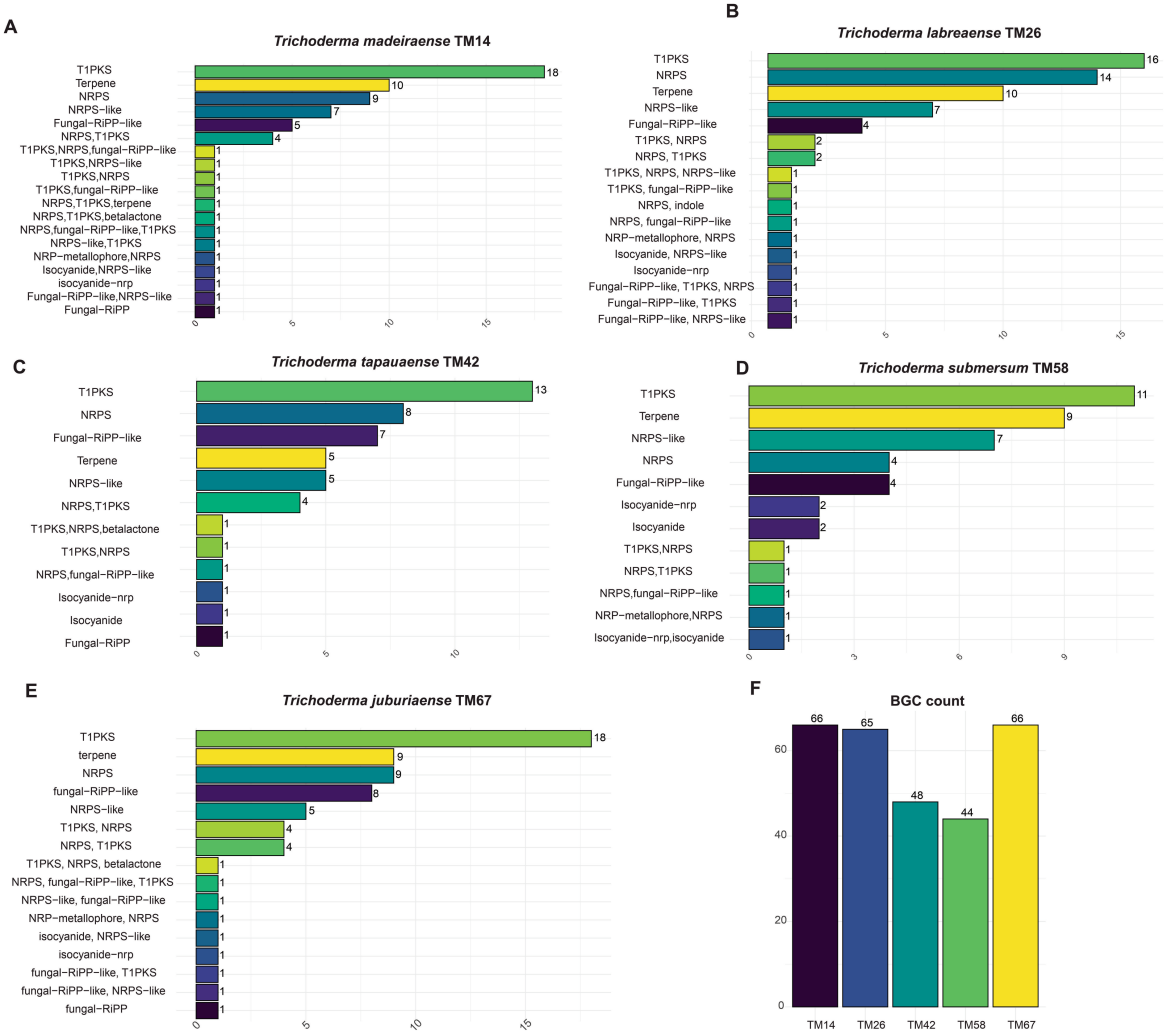
Biosynthetic gene cluster (BGC) composition across *Trichoderma* species. **(A–E)** Distribution of BGC classes identified in *Trichoderma madeiraense* TM14, *T. labreaense* TM26, *T. tapauaense* TM42, *T. submersum* TM58, and *T. juburiaense* TM67. **(F)** Total BGC count per strain.

The biosynthetic potential was assessed through genome mining for biosynthetic gene clusters (BGCs), revealing significant variation in both abundance and diversity among the new species (Figures 12A–E). The most prevalent BGC classes across all strains were Type I Polyketide Synthase (T1PKS) and Non-Ribosomal Peptide Synthetase (NRPS), underscoring their central role in secondary metabolism. Notably, *T. labreaense* TM26 exhibited the highest number of NRPS clusters (14), suggesting a strong capacity for peptide biosynthesis, while *T. madeiraense* TM14 and *T. juburiaense* TM67 harbored the most polyketide-related clusters (18). Furthermore, *T. submersum* TM58 displayed an increased number of isocyanide-associated clusters (2), whereas other species contained only one. Among all analyzed strains, *T. juburiaense* TM67 and *T. madeiraense* TM14 possessed the highest total number of BGCs (66), whereas *T. submersum* TM58 had the lowest (44) (Figure 12F).

**Figure 13:**
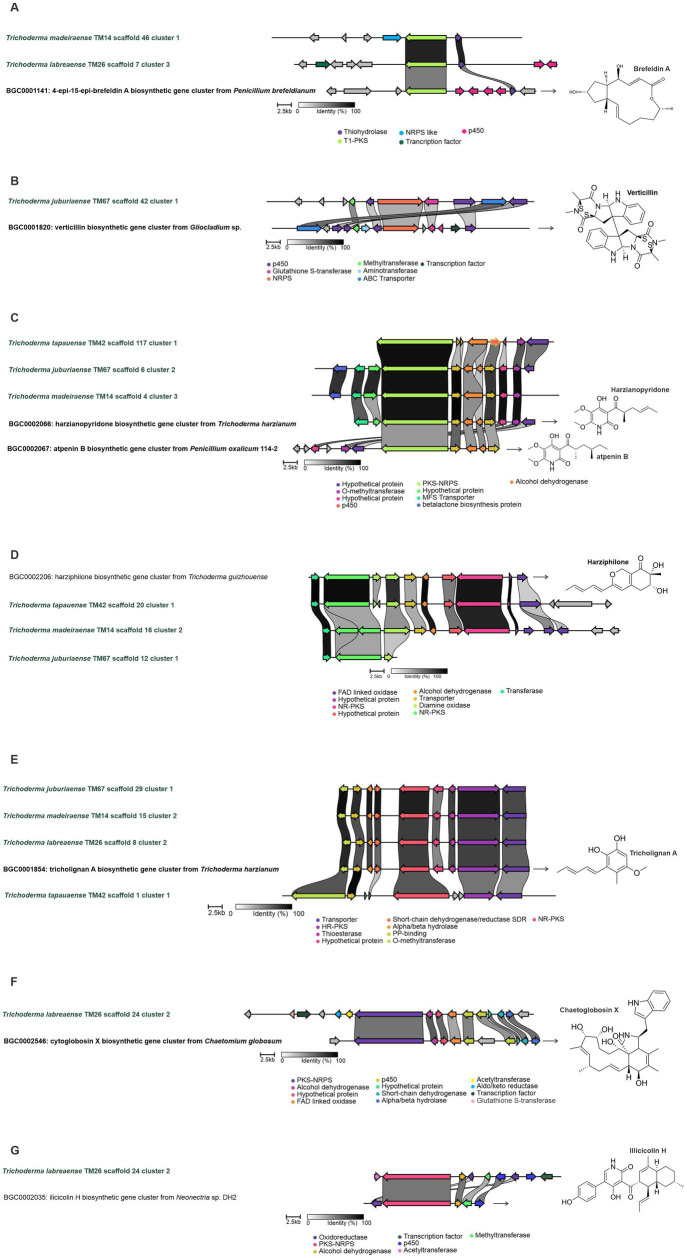
Synteny analysis of biosynthetic gene clusters (BGCs) associated with biologically active compounds identified in the genome of the new *Trichoderma* species. **(A)** Genes related to the Brefeldin A BGC (*BGC0001141*) from *Penicillium brefeldianum* in *Trichoderma madeiraense* TM14 and *Trichoderma labreaense* TM26. **(B)** Genes related to the Verticillin BGC (*BGC0001820*) from *Gliocladium* sp. in *T. juburiaense* TM67. **(C)** Genes related to the Harzianopyridone BGC (*BGC0002066*) from *Trichoderma harzianum* in *T. juburiaense* TM67 and *T. madeiraense* TM14. **(D)** Genes related to the Harziphilone BGC (*BGC0002206*) from *Trichoderma guizhouense* in *T. juburiaense* TM67 and *T. madeiraense* TM14. **(E)** Genes related to the Tricholignan A BGC (*BGC0001854*) from *Trichoderma harzianum* in *T. juburiaense* TM67, *T. madeiraense* TM14, and *T. labreaense* TM26. **(F)** Genes related to the Chaetoglobosin X BGC (*BGC0002546*) from *Chaetomium globosum* in *T. labreaense* TM26. **(G)** Genes related to the Ilicicolin H BGC (*BGC0002035*) from *Neonectria* sp. in *T. labreaense* TM26.

Further analysis identified core genes for macrolide polyketide biosynthesis, including pathways related to brefeldin A-like compounds in *T. madeiraense* TM14 and *T. labreaense* TM26. The presence of two non-homologous P450 enzymes suggests distinct structural modifications, such as hydroxylation reactions (Figure 13A). In *T. juburiaense* TM67, all biosynthetic genes required for verticillin production were detected, indicating its potential to synthesize verticillin or structurally related epipolythiodioxopiperazine alkaloids (Figure 13B).

Clusters responsible for the biosynthesis of harzianopyridone and harziphilone were identified in *T. madeiraense* TM14, *T. tapauaense* TM42, and *T. juburiaense* TM67 (Figures 13C,D). These species, along with *T. labreaense* TM26, also contained all genes necessary for tricholignan biosynthesis (Figure 13E), a pathway previously characterized in *T. harzianum*. Furthermore, *T. labreaense* TM26 harbored a complete set of genes for chaetoglobosin biosynthesis (Figure 13F), as well as those involved in Ilicicolin production (Figure 13G). Notably, this strain exhibited non-homologous genes encoding biosynthetic enzymes, such as P450 enzymes and acetyltransferases within the Ilicicolin BGC, suggesting potential structural modifications that could yield novel analogs.

## 4 Discussion

The Amazon basin encompasses blackwater, whitewater, and clearwater rivers; however, all *Trichoderma* strains were obtained from whitewater rivers (Juruá, Purus, and Madeira). In these rivers, the sediment load is most abundant (Junk, 1984). These receive a large amount of sediments from the Andes region and carry them to the Amazon River, which discharges them in the Atlantic Ocean (McClain and Naiman, 2008). Moreover, the samples were collected in the high-water period, when the microbiota is increased by the bioavailability of substrates acquired from the extensive “várzea” (Amazonian floodplain) (Benner et al., 1995).

The physicochemical conditions recorded at the sampling sites, with pH ranging from 5.5 to 6.0 and water temperatures between 24.0 °C and 32.5 °C (Table 1), are consistent with the well-documented characteristics of Amazonian white-water rivers, which are typified by near-neutral pH and nutrient-rich sediments derived from Andean erosion (Ríos-Villamizar et al., 2013; McClain and Naiman, 2008). These conditions align closely with the optimal pH range reported for *Trichoderma* growth and activity (pH 4–6), within which sporulation rates, mycoparasitic activity, and volatile antifungal metabolite production are maximized (Cai and Druzhinina, 2021; Ali et al., 2018). The coincidence between the physicochemical profile of white-water river sediments and the optimal growth conditions for *Trichoderma* may partly explain the successful isolation and diversity of this genus from these environments and supports the ecological relevance of white-water Amazonian sediments as a favorable niche for *Trichoderma* establishment and diversification.

Among the isolates obtained, *T. lentiforme* was the most frequently recovered species, followed by *T. cyanodichotomus* and *T. afroharzianum*. To our knowledge, this study reports the first occurrence of *T. cyanodichotomus* and *T. awajun* in Brazil. *T. cyanodichotomus* has been previously reported only in China, where it exhibited antagonistic activity against nematodes and fungal plant pathogens (Li et al., 2018; Zhou et al., 2020), while *T. awajun* has been documented exclusively in Argentina. Conversely, *T. lentiforme* and *T. afroharzianum* are widely distributed in tropical South America, with previous records in Colombia, Argentina, Peru, and Brazil (Chaverri et al., 2015; Inglis et al., 2020; Silva et al., 2021). It is important to emphasize that *T. lentiforme* was isolated in all sediments of Amazonian rivers studied in this work. Many strains of *T. afroharzianum* are recognized for their biocontrol activity against several phytopathogens. Here, we describe *T. tapauaense* sp. nov., which forms a sister clade with *T. afroharzianum*, and present its potential as a biocontrol agent, as evidenced by *in vitro* antagonistic activity against different *Colletotrichum* species.

Interestingly, *T. juburiaense* formed a sister group to the *Trichoderma* species, which was considered rare due to the few isolates reported as *T. hainanense* and *T. pyramidale* (Cai and Druzhinina, 2021). These data demonstrated that Amazon ecosystems can be exploited to unconventional *Trichoderma* strains obtention and bioprospection of new genetic resources.

Other studies have isolated *Trichoderma* in the Amazonia biome. In Colombian Amazonia, a set of 107 strains was identified by phylogenetic analysis using the *tef1-α* barcode; these strains were classified as *T. harzianum*, *T. spirale*, *T. koningiopsis*, and a new species, *T. strigosellum*, was proposed (López-Quintero et al., 2013). In Peruvian Amazonia, a set of 199 isolates was tested against *Moniliophthora roreri*, *the* causal agent of frosty pod rot in *Theobroma cacao*, resulting in strains that inhibited the phytopathogen in 38.99 to 71.9% (Leiva et al., 2020). The *T. theobromicola* (Samuels et al., 2006b) and *T. amazonicum* (Chaverri et al., 2011) were described as new species. In Brazilian Amazonia, the new species *T. ovalisporum* (Holmes et al., 2004) and *T. agriamazonicum* (Sousa et al., 2023) were proposed.

The delimitation of *T. submersum* TM58 as a novel species was based on *tef1-α* phylogenetic placement and morphological evidence, given that *rpb2* sequence data for the *T. stilbohypoxyli* type strain are currently unavailable. Despite this limitation, morphological examination clearly distinguishes the two taxa. Phialides of *T. submersum* (*M* = 8.75 μm) are notably longer than those of *T. stilbohypoxyli* (ca. 7.0 μm; Samuels et al., 2006a), and conidia are likewise longer (M = 4.16 μm vs. 3.4–3.5 μm). These morphological differences are further corroborated by 17 diagnostic nucleotide positions in the *tef1-α* alignment relative to *T. stilbohypoxyli* GJS 96–32 (Supplementary Figure 3). Together, the morphometric and molecular evidence support the recognition of TM58 as a distinct species, despite the absence of a full multilocus framework for this comparison.

Regarding the antagonistic potential of the new species described in this study, all species were able to antagonize the phytopathogen species. Interestingly, *Trichoderma juburiaense* exhibited the best performance in most dual-culture assays and demonstrated great mycoparasitic potential by growing over the phytopathogen. Antagonism by *Trichoderma* can occur through the production of enzymes, secondary metabolites, volatile organic compounds, and mycoparasitism (Woo et al., 2022).

The differences in genomic content and CAZyme profiles found among the *Trichoderma* species examined, such as the elevated GH18 and GH16 annotations in *T. juburiaense* and *T. madeiraense*, respectively, strengthens the notion that genomic variation plays a key role in the functional diversity of enzymes involved in the degradation of fungal cell walls, a critical aspect of mycoparasitism and the biocontrol potential of *Trichoderma* species. This aligns with earlier research suggesting that such diversity is fundamental to the wide-ranging ecological functions and industrial applications of *Trichoderma* species, with mycoparasitism being proposed as the ancestral lifestyle of this genus (Kubicek et al., 2019).

The species *T. gamsii* T6085 and *T. submersum* sp. nov. TM58 exhibited notably lower numbers of CAZymes in general, which can be highlighted with pectin degradation (9 and 13, respectively), suggesting a reduced capacity to act on this substrate. This difference may reflect a specialization or specific adaptation related to the types of biomass these species can efficiently degrade.

The widespread representation of the AA9 family (ranging from 23 to 25 proteins across isolates) suggests its conserved role in cellulose degradation, as this family is known for its involvement in oxidative cleavage of cellulose, an essential step in cellulose breakdown. The increased presence of GH45, particularly in *T. tapauaense* TM42, further supports this species ability to decompose cellulose efficiently, as GH45 enzymes are key in breaking down complex polysaccharides like cellulose.

In lignin degradation, notable expansions of the GH3 and AA3 families were observed, particularly in *T. labreaense* TM26. This suggests that *T. labreaense* may have a specialized ability to degrade lignin, a highly complex and recalcitrant component of plant biomass. Moreover, the presence of multiple proteins with the AA3 + AA3 domain composition in *T. tapauaense* TM42 suggests that this species may have evolved mechanisms to break down lignin and other complex polymers in plant biomass that are absent in closely related species.

The elevated GH16 annotation observed in *T. juburiaense* TM67, a family encoding β-1,3-glucanases associated with fungal cell wall degradation, is consistent with its strong mycoparasitic performance in dual-culture assays. Similarly, the high GH18 counts in *T. labreaense* TM26 suggest a genomic capacity for chitinase production, a key enzyme class in mycoparasitism. However, it should be noted that genomic annotation reflects biosynthetic potential rather than confirmed gene expression or protein activity under the conditions tested. Functional validation through transcriptomic or proteomic approaches will be necessary to establish direct links between CAZyme repertoires and observed antagonistic phenotypes.

Regarding the biosynthetic potential for secondary metabolite production, we identified clusters associated with valuable and bioactive compounds, including molecules with antiviral, antifungal, antibiotic, and cytotoxic activities. In particular, the biosynthetic gene cluster (BGC) for the production of verticillins was identified and characterized in the genome of *Trichoderma juburiaense* sp. nov. Verticillins and their analogs exhibit a broad range of biological activities. For instance, verticillin G is a potent antibiotic against methicillin-resistant and quinolone-resistant *Staphylococcus aureus*, with a minimum inhibitory concentration (MIC) of 3–10 μg/mL (Zheng et al., 2007).

Furthermore, new analogs based on verticillin H have been screened for anticancer activity, and all tested compounds exhibited IC50 values in the nanomolar range (Amrine et al., 2021). To date, 27 verticillin analogs have been reported, with the main producers being fungi of the *Bionectriaceae* family, particularly within the genus *Clonostachys* (Pierre et al., 2024). Interestingly, no verticillins have been reported in *Trichoderma* until now, suggesting that this biosynthetic cluster may be silent under laboratory conditions.

Another BGC detected in *Trichoderma labreaense* sp. nov. was associated with Ilicicolin H. This molecule exhibits potent and broad-spectrum antifungal activity, effectively inhibiting *Candida* spp., *Aspergillus fumigatus*, and *Cryptococcus* spp., with sub-μg/mL MICs. It acts via a novel mode of action by inhibiting mitochondrial cytochrome bc1 reductase, with an IC50 of 2–3 ng/mL. In addition to its antifungal properties, its analog, Ilicicolin J, has demonstrated anti-biofilm activity against *Staphylococcus aureus* by disrupting the exopolysaccharides in the biofilm matrix (Tang et al., 2023). Interestingly, although *T. labreaense* possesses all the genes necessary for Ilicicolin H production, this strain also harbors additional acetyltransferase and P450 enzymes. This suggests the potential for the biosynthesis of novel Ilicicolin analogs, expanding its chemical diversity and possible bioactivities.

Furthermore, a cluster with a gene composition similar to that of the Ilicicolin H BGC was identified in *Trichoderma reesei* QM6a. Heterologous expression of this cluster in *Aspergillus nidulans* led to the production of Ilicicolin H (Shenouda et al., 2021). Interestingly, this cluster remained silent under laboratory conditions, aligning with previous reports that Ilicicolin H has not been naturally detected in *Trichoderma* species without targeted refactoring of the cluster.

In the genome of *Trichoderma labreaense*, we also identified a cluster related to chaetoglobosin production. These compounds are recognized for their antifungal and cytotoxic activities and can act as elicitors of resistance by inducing oxidative bursts in plants (Soytong et al., 2001). Chaetoglobosin A (ChA) preferentially induces apoptosis in chronic lymphocytic leukemia (CLL) cells by targeting filamentous actin, leading to cell-cycle arrest, inhibition of membrane ruffling, and reduced cell migration (Knudsen et al., 2014). Chaetoglobosin P disrupts filamentous actin polymerization by interfering with the capping process, with a potential self-resistance mechanism involving twinfilin-1. In *Cryptococcus neoformans*, the absence of twinfilin-1 increases sensitivity to chaetoglobosin P, thereby enhancing the antifungal effects of amphotericin B and caspofungin (Perlatti et al., 2020). The genome of *Trichoderma labreaense* sp. Nov contains all the necessary genes for the biosynthesis of cytochalasins, and its biosynthetic gene cluster (BGC) includes additional enzymes, such as an acetyltransferase and an aldo-keto reductase, which may contribute to structural modifications of cytochalasin molecules.

The identification of biosynthetic gene clusters (BGCs) for brefeldin A production in *Trichoderma labreaense* sp. nov. and *T. madeiraense* sp. nov. highlights the potential of these species to synthesize this biologically significant macrolide. Brefeldin A is a well-known inhibitor of protein trafficking with antiviral, antifungal, and anticancer properties (Seehafer et al., 2013; Farias et al., 2019). Interestingly, the BGC found in *T. labreaense* harbors non-homologous P450 monooxygenases, suggesting the possibility of unique structural modifications that could lead to novel analogs with distinct bioactivities. Moreover, in *T. madeiraense*, the cluster includes an NRPS-like (non-ribosomal peptide synthetase-like) gene, which is not typically associated with brefeldin A biosynthesis. This unusual genomic feature suggests the potential to produce hybrid molecules or new variations.

The species *T. juburiaense*, *T. tapauaense*, *T. labreaense*, and *T. madeiraense* possess BGCs previously characterized in other *Trichoderma* species, such as those responsible for harziphilone, harzianopyridone, and tricholignan A biosynthesis. Harziphilone and its analog fleephilone, initially reported in *Trichoderma harzianum*, are inhibitors of the HIV-1 Rev-RRE interaction, with harziphilone also displaying cytotoxic activity against murine tumor cells at 38 μM (Qian-Cutrone et al., 1996). Harzianopyridone is a potent inhibitor of mitochondrial complex II and has demonstrated strong antiviral activity against Zika virus (ZIKV). It effectively suppresses ZIKV replication (EC50: 0.46–2.63 μM) with low cytotoxicity and directly inhibits the viral RNA-dependent RNA polymerase (RdRp), reducing viral protein expression and protecting cells from infection (Bat-Erdene et al., 2020; Zhang et al., 2024). Tricholignan A, produced by *Trichoderma harzianum* T-22, is a redox-active ortho-hydroquinone that reduces Fe(III), potentially aiding plant growth under iron-deficient conditions (Chen et al., 2019).

## 5 Conclusion

This study revealed the rich diversity of the genus *Trichoderma* in sediments from Amazonian rivers, leading to the characterization of 44 isolates belonging to 14 distinct species, including the description of five new species (*T. madeiraense*, *T. labreaense*, *T. tapauaense*, *T. submersum*, and *T. juburiaense*) and the first record of *T. cyanodichotomus* and *T. awajun* in Brazil. The widespread occurrence of *T. lentiforme* across all sampled sediments highlights its adaptability to Amazonian environments. *In vitro* antagonistic assays showed that all newly described species exhibit biocontrol activity against major phytopathogens, with *T. juburiaense* TM67 standing out for its strong antagonistic potential, significantly inhibiting the growth of various *Colletotrichum* species and other pathogens, including *Bipolaris oryzae*.

Genomic analyses provided deeper insights into the biotechnological potential of these new species. CAZyme profiling revealed a diverse array of enzymes involved in the degradation of plant and fungal cell wall components, with a notable abundance of glycoside hydrolases from families GH16, GH18, and GH72, associated with mycoparasitism and fungal cell wall breakdown. High representation of enzymes like AA9 and GH45 supports the strong cellulose-degrading capabilities of these species, while variability in enzymatic profiles among isolates suggests ecological specialization and adaptation.

Genome mining for biosynthetic gene clusters (BGCs) highlighted the metabolic versatility of these species, revealing their capacity to produce a broad spectrum of bioactive secondary metabolites. BGCs associated with the synthesis of compounds such as verticillins, ilicicolins, chaetoglobosins, harzianopyridone, and brefeldin A were identified, many of which have known antifungal, antibacterial, and cytotoxic properties. Notably, *T. juburiaense* TM67 harbored the complete BGC for verticillin biosynthesis. *T. labreaense* TM26 contained BGCs for ilicicolin H and chaetoglobosin production, indicating its potential as a source of potent antifungal and pharmacologically active molecules.

This study underscores the Amazonian environment as a reservoir of microorganisms with significant biotechnological potential. By integrating phylogenetic, morphological, functional, and genomic analyses, we not only described new *Trichoderma* species but also identified promising strains for biocontrol applications and biosynthetic potential for valuable secondary production.

## Data Availability

The datasets presented in this study can be found in online repositories. The names of the repository/repositories and accession number(s) can be found in the article/Supplementary material.

## Footnotes

1
www.mycobank.org

2
https://www.phylo.org/

3
https://usegalaxy.org.au/

4
https://bcb.unl.edu/dbCAN2/

5
https://fungismash.secondarymetabolites.org/
