# Supplementary material for: Isolation and characterization of Trichoderma from Amazonian white-water river sediments with descriptions of five new species and new records from Brazil
Source: Front Microbiol. 2026 May 20;17:1778622. doi: 10.3389/fmicb.2026.1778622 (PMC13229981; doi:10.3389/fmicb.2026.1778622)
Supplement: Supplementary file 1 [file Data_Sheet_1.PDF]

Supplementary Material

1. Supplementary tables

Table S1: Detailed information on the phytopathogens used in this study

Phytopathogen	Strain	Host	Molecular identification	Tissue
Colletotrichum siamense	Coll 2N	*Synedrella nodiflora*	ITS, *tub2*	Leaf
Colletotrichum theobromicola	INPA 1809	*Allium fistulosum*	*act*, *gapdh*, *chs*	Leaf
Colletotrichum spaethianum	INPA 2908	*Allium fistulosum*	ITS, *tub2*, *cam*, *chs*, *act*	Leaf
Colletotrichum scovillei	INPA 2910	*Capsicum chinense*	*gapdh*, *tub2*	Fruit
Colletotrichum sp.	INPA 2973	*Carica papaya*	Morphology	Fruit
Fusarium fabacearum	MCT10621	*Solanum lycopersicum*	tef-1α	Fruit
Pyricularia oryzae	BRM 10.781	*Oryza sativa*	ITS	Leaf
Bipolaris oryzae	BRM 46676	*Oryza sativa*	ITS	Leaf
Rhizoctonia solani	BRM 45111	*Phaseolus vulgaris*	ITS	Root

Table S2: **BUSCO Assessment summary**

Genome	Source	Complete BUSCOs (C)	Single-copy (S)	Duplicated (D)	Fragmented (F)	Missing (M)	Internal stop codons (E)
**TM14**	This study	99.1%	99.0%	0.1%	0.2%	0.7%	4.1%
**TM26**	This study	99.0%	98.8%	0.2%	0.2%	0.8%	4.2%
**TM42**	This study	99.1%	99.0%	0.1%	0.1%	0.8%	4.4%
**TM58**	This study	99.0%	99.0%	0.0%	0.2%	0.8%	3.7%
**TM67**	This study	98.9%	98.7%	0.2%	0.2%	0.9%	3.5%
***T. afroharzianum* Th6**	NCBI RefSeq	99.0%	98.8%	0.2%	0.1%	0.9%	4.5%
***T. aggressivum* CBS100526**	NCBI RefSeq	98.9%	98.8%	0.1%	0.2%	0.9%	3.9%
***T. gamsii* T6085**	NCBI RefSeq	98.7%	98.6%	0.1%	0.3%	1.0%	3.3%
***T. guizhouense* NJAU4742**	NCBI RefSeq	99.0%	98.8%	0.2%	0.2%	0.8%	4.2%
***T. virens* Gv29-8**	NCBI RefSeq	99.0%	98.8%	0.2%	0.2%	0.8%	3.9%

Table S3. Genome assembly statistics of *Trichoderma* isolates and reference genomes used in this study

Species/Strain	Size (Mb)	Contigs	N50 (kb)	N90 (kb)	Largest contig (Mb)	GC (%)	Accession/Source
***T. guizhouense* NJAU4742**	38.3	63	2,415	736	3.57	49.6	LVVK00000000.1
***T. virens* Gv29-8**	39.0	93	1,837	329	3.46	49.2	GCF_000170995.1
***T. afroharzianum* Th6**	41.6	142	1,103	220	3.80	46.7	JBBLXW000000000
***T gamsii* T6085**	37.9	172	697	179	1.83	48.9	JPDN00000000
***Trichoderma aggressivum* CBS100526**	38.9	295	580	163	2.23	49.1	JAWRVG000000000
**TM26**	40.2	363	1,865	454	3.90	48.4	JBVHAO000000000
**TM14**	39.8	365	1,450	361	3.12	47.9	JBVHAN000000000
**TM67**	39.4	317	871	303	2.30	48.9	JBVHAR000000000
**TM58**	38.1	856	619	107	2.26	46.7	JBVHAQ000000000
**TM42**	43.4	1,439	274	21	1.10	43.2	JBVHAP000000000

2. Supplementary Figures

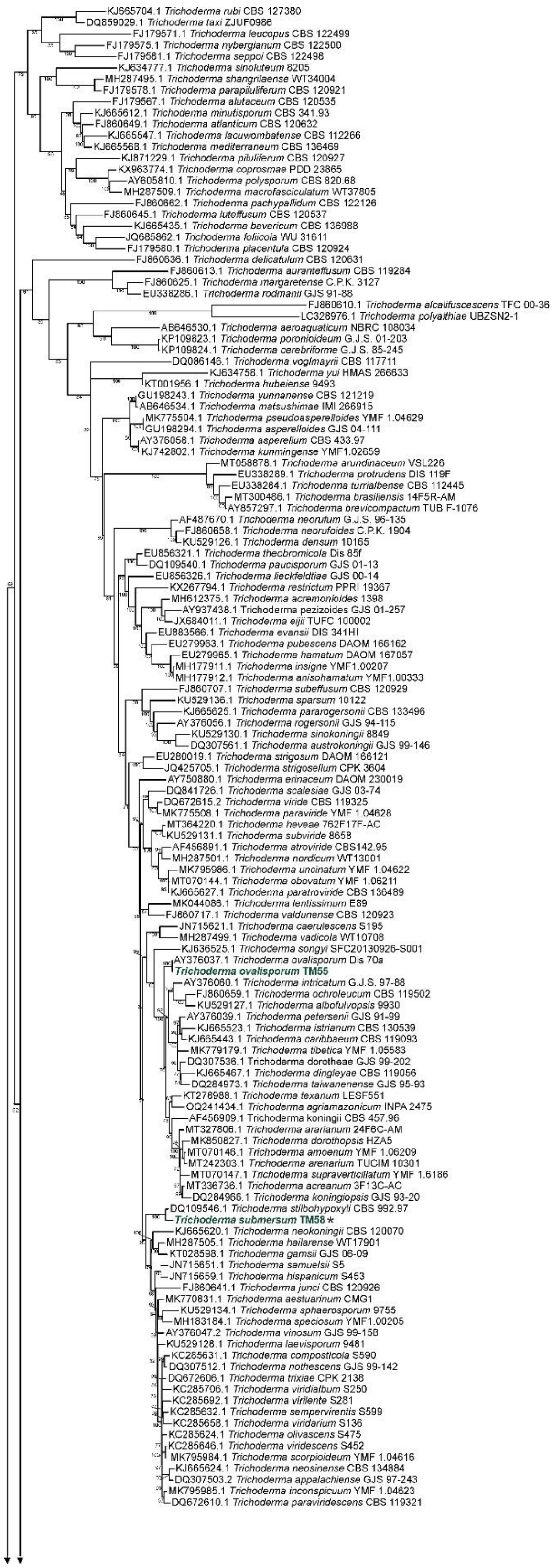

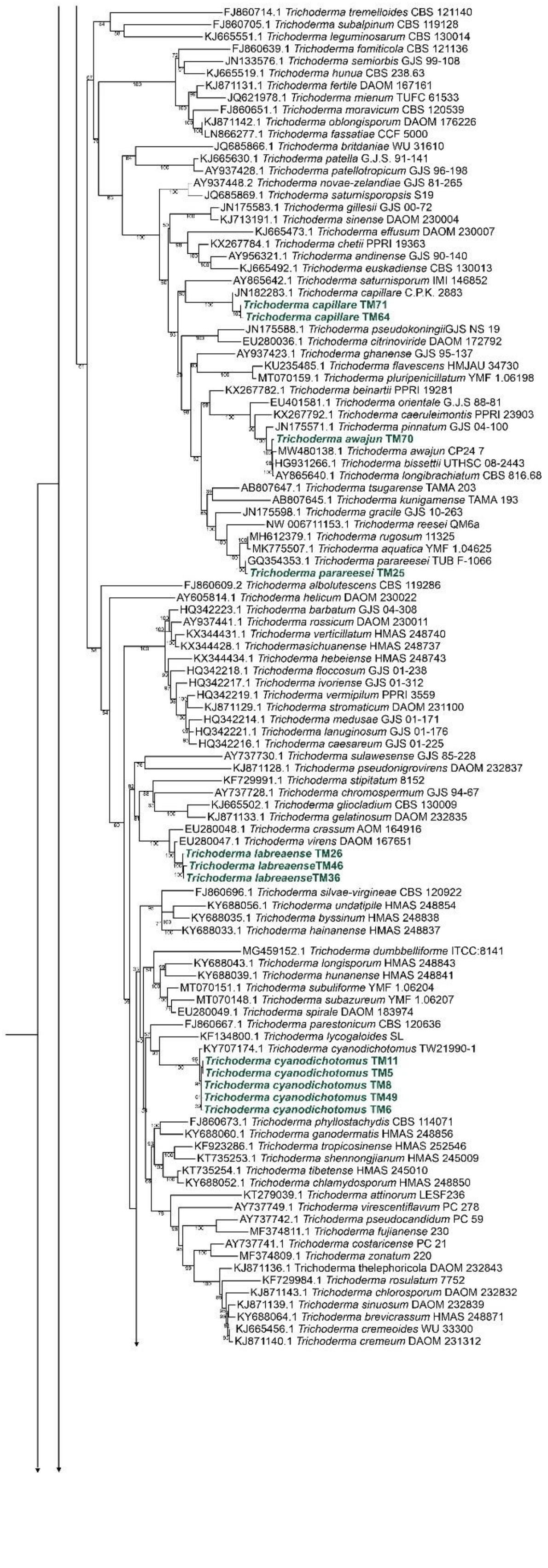

FJ860714.1 Trichoderma tremelloides CBS 121140
FJ860705.1 Trichoderma subalpinum CBS 119128
KJ665551.1 Trichoderma leguminosarum CBS 130014
FJ860639.1 Trichoderma fomiticola CBS 121136
JN133576.1 Trichoderma semiorbis GJS 99-108
KJ665519.1 Trichoderma hunua CBS 238.63
KJ871131.1 Trichoderma fertile DAOM 167161
JQ621978.1 Trichoderma mienum TUFC 61533
FJ860651.1 Trichoderma moravicum CBS 120539
KJ871142.1 Trichoderma oblongisporum DAOM 176226
LN866277.1 Trichoderma fassatiae CCF 5000
JQ685866.1 Trichoderma britdaniae WU 31610
KJ665630.1 Trichoderma patella G.J.S. 91-141
AY937428.1 Trichoderma patellotropicum GJS 96-198
AY937448.2 Trichoderma novae-zelandiae GJS 81-265
JQ685869.1 Trichoderma saturnisporopsis S19
JN175583.1 Trichoderma gillesii GJS 00-72
KJ713191.1 Trichoderma sinense DAOM 230004
KJ665473.1 Trichoderma effusum DAOM 230007
KX267784.1 Trichoderma chetii PPRI 19363
AY956321.1 Trichoderma andinense GJS 90-140
KJ665492.1 Trichoderma euskadiense CBS 130013
AY865642.1 Trichoderma saturnisporum IMI 146852
JN182283.1 Trichoderma capillare C.P.K. 2883
Trichoderma capillare TM71
Trichoderma capillare TM64
JN175588.1 Trichoderma pseudokoningiiGJS NS 19
EU280036.1 Trichoderma citrinoviride DAOM 172792
AY937423.1 Trichoderma ghanense GJS 95-137
KU235485.1 Trichoderma flavescens HMJAU 34730
MT070159.1 Trichoderma pluripenicillatum YMF 1.06198
KX267782.1 Trichoderma beinartii PPRI 19281
EU401581.1 Trichoderma orientale G.J.S 88-81
KX267792.1 Trichoderma caeruleimontis PPRI 23903
JN175571.1 Trichoderma pinnatum GJS 04-100
Trichoderma awajun TM70
MW480138.1 Trichoderma awajun CP24 7
HG931266.1 Trichoderma bissettii UTHSC 08-2443
AY865640.1 Trichoderma longibrachiatum CBS 816.68
AB807647.1 Trichoderma tsugarense TAMA 203
AB807645.1 Trichoderma kunigamense TAMA 193
JN175598.1 Trichoderma gracile GJS 10-263
NW 006711153.1 Trichoderma reesei QM6a
MH612379.1 Trichoderma rugosum 11325
MK775507.1 Trichoderma aquatica YMF 1.04625
GQ354353.1 Trichoderma parareesei TUB F-1066
Trichoderma parareesei TM25
FJ860609.2 Trichoderma albolutescens CBS 119286
AY605814.1 Trichoderma helicum DAOM 230022
HQ342223.1 Trichoderma barbatum GJS 04-308
AY937441.1 Trichoderma rossicum DAOM 230011
KX344431.1 Trichoderma verticillatum HMAS 248740
KX344428.1 Trichodermasichuanense HMAS 248737
KX344434.1 Trichoderma hebeiense HMAS 248743
HQ342218.1 Trichoderma floccosum GJS 01-238
HQ342217.1 Trichoderma ivoriense GJS 01-312
HQ342219.1 Trichoderma vermipilum PPRI 3559
KJ871129.1 Trichoderma stromaticum DAOM 231100
HQ342214.1 Trichoderma medusae GJS 01-171
HQ342221.1 Trichoderma lanuginosum GJS 01-176
HQ342216.1 Trichoderma caesareum GJS 01-225
AY737730.1 Trichoderma sulawesense GJS 85-228
KJ871128.1 Trichoderma pseudonigrovirens DAOM 232837
KF729991.1 Trichoderma stipitatum 8152
AY737728.1 Trichoderma chromospermum GJS 94-67
KJ665502.1 Trichoderma gliocladium CBS 130009
KJ871133.1 Trichoderma gelatinosum DAOM 232835
EU280048.1 Trichoderma crassum AOM 164916
EU280047.1 Trichoderma virens DAOM 167651
Trichoderma labreaense TM26
Trichoderma labreaenseTM46
Trichoderma labreaenseTM36
FJ860696.1 Trichoderma silvae-virgineae CBS 120922
KY688056.1 Trichoderma undatipile HMAS 248854
KY688035.1 Trichoderma byssinum HMAS 248838
KY688033.1 Trichoderma hainanense HMAS 248837
MG459152.1 Trichoderma dumbbelliforme ITCC:8141
KY688043.1 Trichoderma longisporum HMAS 248843
KY688039.1 Trichoderma hunanense HMAS 248841
MT070151.1 Trichoderma subuliforme YMF 1.06204
MT070148.1 Trichoderma subazureum YMF 1.06207
EU280049.1 Trichoderma spirale DAOM 183974
FJ860667.1 Trichoderma parestonicum CBS 120636
KF134800.1 Trichoderma lycogaloides SL
KY707174.1 Trichoderma cyanodichotomus TW21990-1
Trichoderma cyanodichotomus TM11
Trichoderma cyanodichotomus TM5
Trichoderma cyanodichotomus TM8
Trichoderma cyanodichotomus TM49
Trichoderma cyanodichotomus TM6
FJ860673.1 Trichoderma phyllostachydis CBS 114071
KY688060.1 Trichoderma ganodermatis HMAS 248856
KF923286.1 Trichoderma tropicosinense HMAS 252546
KT735253.1 Trichoderma shennongjianum HMAS 245009
KT735254.1 Trichoderma tibetense HMAS 245010
KY688052.1 Trichoderma chlamydosporum HMAS 248850
KT279039.1 Trichoderma attinorum LESF236
AY737749.1 Trichoderma virescentiflavum PC 278
AY737742.1 Trichoderma pseudocandidum PC 59
MF374811.1 Trichoderma fujianense 230
AY737741.1 Trichoderma costaricense PC 21
MF374809.1 Trichoderma zonatum 220
KJ871136.1 Trichoderma thelephoricola DAOM 232843
KF729984.1 Trichoderma rosulatum 7752
KJ871143.1 Trichoderma chlorosporum DAOM 232832
KJ871139.1 Trichoderma sinuosum DAOM 232839
KY688064.1 Trichoderma brevicrassum HMAS 248871
KJ665456.1 Trichoderma cremeoides WU 33300
KJ871140.1 Trichoderma cremeum DAOM 231312

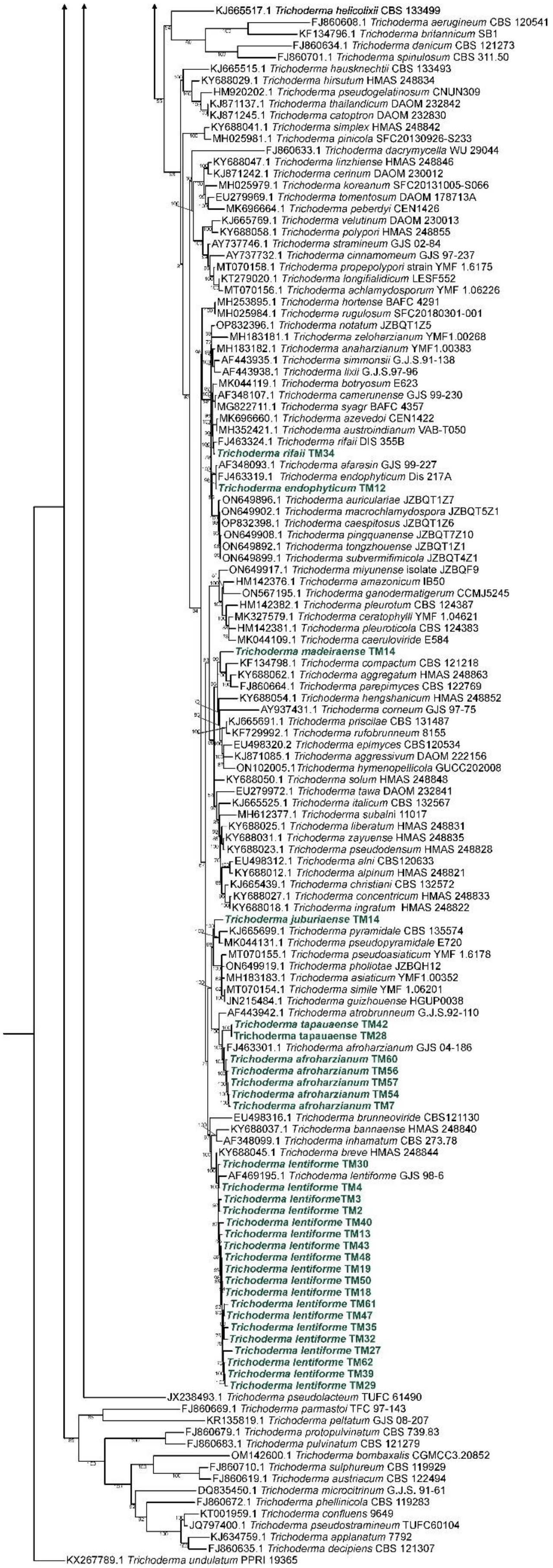
KJ665517.1 Trichoderma helicolixii CBS 133499
FJ860608.1 Trichoderma aerugineum CBS 120541
KF134796.1 Trichoderma britannicum SB1
FJ860634.1 Trichoderma danicum CBS 121273
FJ860701.1 Trichoderma spinulosum CBS 311.50
KJ665515.1 Trichoderma hausknechtii CBS 133493
KY688029.1 Trichoderma hirsutum HMAS 248834
HM920202.1 Trichoderma pseudogelatinosum CNUN309
KJ871137.1 Trichoderma thailandicum DAOM 232842
KJ871245.1 Trichoderma catoptron DAOM 232830
KY688041.1 Trichoderma simplex HMAS 248842
MH025981.1 Trichoderma pinicola SFC20130926-S233
FJ860633.1 Trichoderma dacrymycella WU 29044
KY688047.1 Trichoderma linzhiense HMAS 248846
KJ871242.1 Trichoderma cerinum DAOM 230012
MH025979.1 Trichoderma koreanum SFC20131005-S066
EU279969.1 Trichoderma tomentosum DAOM 178713A
MK696664.1 Trichoderma peberdyi CEN1426
KJ665769.1 Trichoderma velutinum DAOM 230013
KY688058.1 Trichoderma polypori HMAS 248855
AY737746.1 Trichoderma stramineum GJS 02-84
AY737732.1 Trichoderma cinnamomeum GJS 97-237
MT070158.1 Trichoderma propepolypori strain YMF 1.6175
KT279020.1 Trichoderma longifialidicum LESF552
MT070156.1 Trichoderma achlamydosporum YMF 1.06226
MH253895.1 Trichoderma hortense BAFC 4291
MH025984.1 Trichoderma rugulosum SFC20180301-001
OP832396.1 Trichoderma notatum JZBQT1Z5
MH183181.1 Trichoderma zeloharzianum YMF1.00268
MH183182.1 Trichoderma anaharzianum YMF1.00383
AF443935.1 Trichoderma simmonsii G.J.S.91-138
AF443938.1 Trichoderma lixii G.J.S.97-96
MK044119.1 Trichoderma botryosum E623
AF348107.1 Trichoderma camerunense GJS 99-230
MG822711.1 Trichoderma syagr BAFC 4357
MK696660.1 Trichoderma azevedoi CEN1422
MH352421.1 Trichoderma austroindianum VAB-T050
FJ463324.1 Trichoderma rifaii DIS 355B
Trichoderma rifaii TM34
AF348093.1 Trichoderma afarasin GJS 99-227
FJ463319.1 Trichoderma endophyticum Dis 217A
Trichoderma endophyticum TM12
ON649896.1 Trichoderma auriculariae JZBQT1Z7
ON649902.1 Trichoderma macrochlamydospora JZBQT5Z1
OP832398.1 Trichoderma caespitosus JZBQT1Z6
ON649908.1 Trichoderma pingquanense JZBQT7Z10
ON649892.1 Trichoderma tongzhouense JZBQT1Z1
ON649899.1 Trichoderma subvermifimicola JZBQT4Z1
ON649917.1 Trichoderma miyunense isolate JZBQF9
HM142376.1 Trichoderma amazonicum IB50
ON567195.1 Trichoderma ganodermatigerum CCMJ5245
HM142382.1 Trichoderma pleurotum CBS 124387
MK327579.1 Trichoderma ceratophylli YMF 1.04621
HM142381.1 Trichoderma pleuroticola CBS 124383
MK044109.1 Trichoderma caeruloviride E584
Trichoderma madeiraense TM14
KF134798.1 Trichoderma compactum CBS 121218
KY688062.1 Trichoderma aggregatum HMAS 248863
FJ860664.1 Trichoderma parepimyces CBS 122769
KY688054.1 Trichoderma hengshanicum HMAS 248852
AY937431.1 Trichoderma corneum GJS 97-75
KJ665691.1 Trichoderma priscilae CBS 131487
KF729992.1 Trichoderma rufobrunneum 8155
EU498320.2 Trichoderma epimyces CBS120534
KJ871085.1 Trichoderma aggressivum DAOM 222156
ON102005.1Trichoderma hymenopellicola GUCC202008
KY688050.1 Trichoderma solum HMAS 248848
EU279972.1 Trichoderma tawa DAOM 232841
KJ665525.1 Trichoderma italicum CBS 132567
MH612377.1 Trichoderma subalni 11017
KY688025.1 Trichoderma liberatum HMAS 248831
KY688031.1 Trichoderma zayuense HMAS 248835
KY688023.1 Trichoderma pseudodensum HMAS 248828
EU498312.1 Trichoderma alni CBS120633
KY688012.1 Trichoderma alpinum HMAS 248821
KJ665439.1 Trichoderma christiani CBS 132572
KY688027.1 Trichoderma concentricum HMAS 248833
KY688018.1 Trichoderma ingratum HMAS 248822
Trichoderma juburiaense TM14
KJ665699.1 Trichoderma pyramidale CBS 135574
MK044131.1 Trichoderma pseudopyramidale E720
MT070155.1 Trichoderma pseudoasiaticum YMF 1.6178
ON649919.1 Trichoderma pholiotae JZBQH12
MH183183.1 Trichoderma asiaticum YMF1.00352
MT070154.1 Trichoderma simile YMF 1.06201
JN215484.1 Trichoderma guizhouense HGUP0038
AF443942.1 Trichoderma atrobrunneum G.J.S.92-110
Trichoderma tapauaense TM42
Trichoderma tapauaense TM28
FJ463301.1 Trichoderma afroharzianum GJS 04-186
Trichoderma afroharzianum TM60
Trichoderma afroharzianum TM56
Trichoderma afroharzianum TM57
Trichoderma afroharzianum TM54
Trichoderma afroharzianum TM7
EU498316.1 Trichoderma brunneoviride CBS121130
KY688037.1 Trichoderma bannaense HMAS 248840
AF348099.1 Trichoderma inhamatum CBS 273.78
KY688045.1 Trichoderma breve HMAS 248844
Trichoderma lentiforme TM30
AF469195.1 Trichoderma lentiforme GJS 98-6
Trichoderma lentiforme TM4
Trichoderma lentiformeTM3
Trichoderma lentiforme TM2
Trichoderma lentiforme TM40
Trichoderma lentiforme TM13
Trichoderma lentiforme TM43
Trichoderma lentiforme TM48
Trichoderma lentiforme TM19
Trichoderma lentiforme TM50
Trichoderma lentiforme TM18
Trichoderma lentiforme TM61
Trichoderma lentiforme TM47
Trichoderma lentiforme TM35
Trichoderma lentiforme TM32
Trichoderma lentiforme TM27
Trichoderma lentiforme TM62
Trichoderma lentiforme TM39
Trichoderma lentiforme TM29
JX238493.1 Trichoderma pseudolacteum TUFC 61490
FJ860669.1 Trichoderma parmastoi TFC 97-143
KR135819.1 Trichoderma peltatum GJS 08-207
FJ860679.1 Trichoderma protopulvinatum CBS 739.83
FJ860683.1 Trichoderma pulvinatum CBS 121279
OM142600.1 Trichoderma bombaxalis CGMCC3.20852
FJ860710.1 Trichoderma sulphureum CBS 119929
FJ860619.1 Trichoderma austriacum CBS 122494
DQ835450.1 Trichoderma microcitrinum G.J.S. 91-61
FJ860672.1 Trichoderma phellinicola CBS 119283
KT001959.1 Trichoderma confluens 9649
JQ797400.1 Trichoderma pseudostramineum TUFC60104
KJ634759.1 Trichoderma applanatum 7792
FJ860635.1 Trichoderma decipiens CBS 121307
KX267789.1 Trichoderma undulatum PPRI 19365

SI figure 1: Phylogram based on Maximum likelihood analysis of *tef1*-α gene. Strains highlighted in green are isolated in this study. The numbers indicated the bootstrap values.

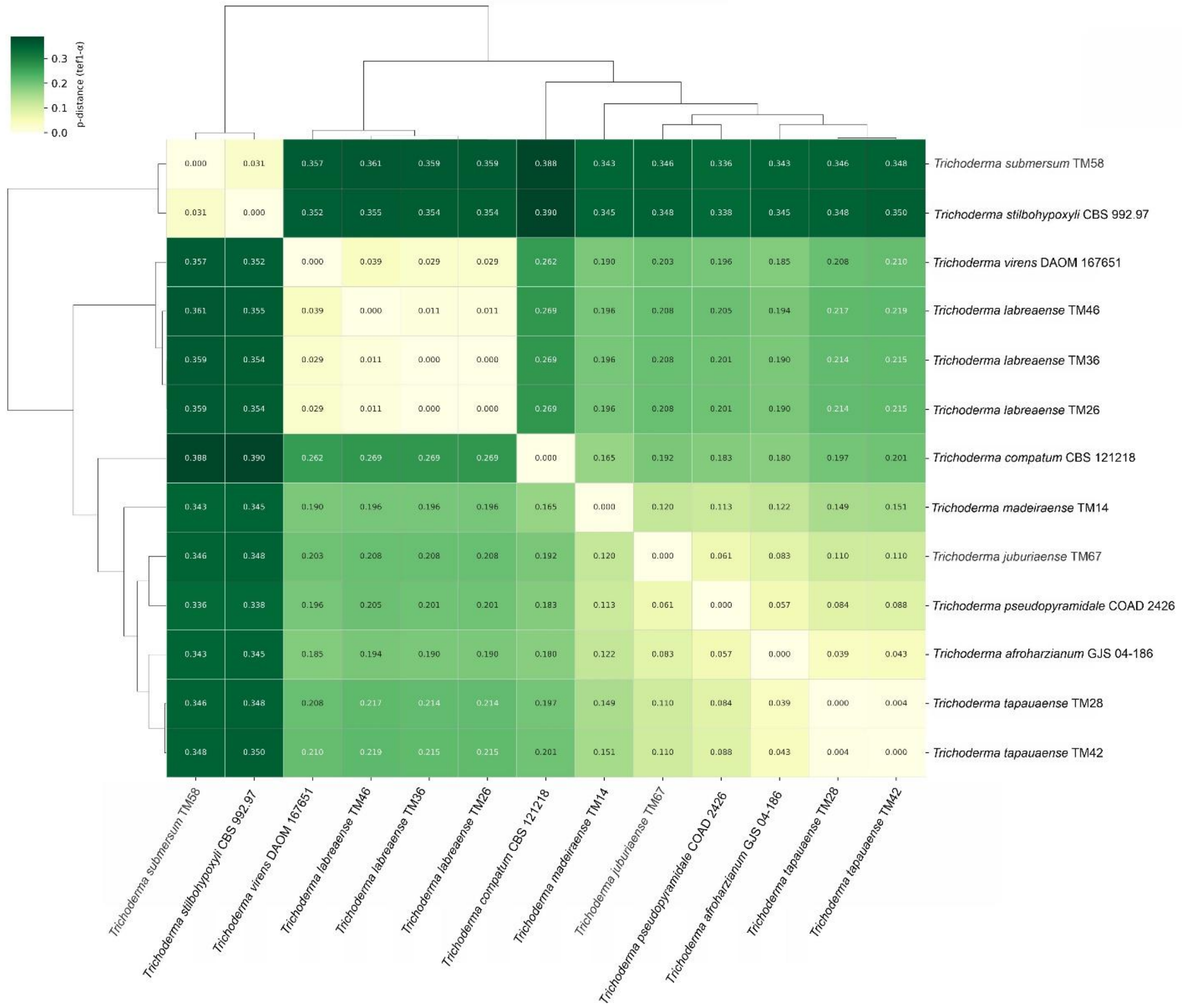

SI figure 2. Pairwise genetic distances based on *tef1-α* partial sequences among novel *Trichoderma* species described in this study and their closest known relatives. Distance values represent p-distances calculated using the identity model. Hierarchical clustering (UPGMA, Euclidean metric) is shown as dendrograms on both axes. Color intensity reflects the magnitude of genetic divergence, ranging from low (yellow) to high (dark green). All sequences were aligned using MAFFT prior to distance calculation.

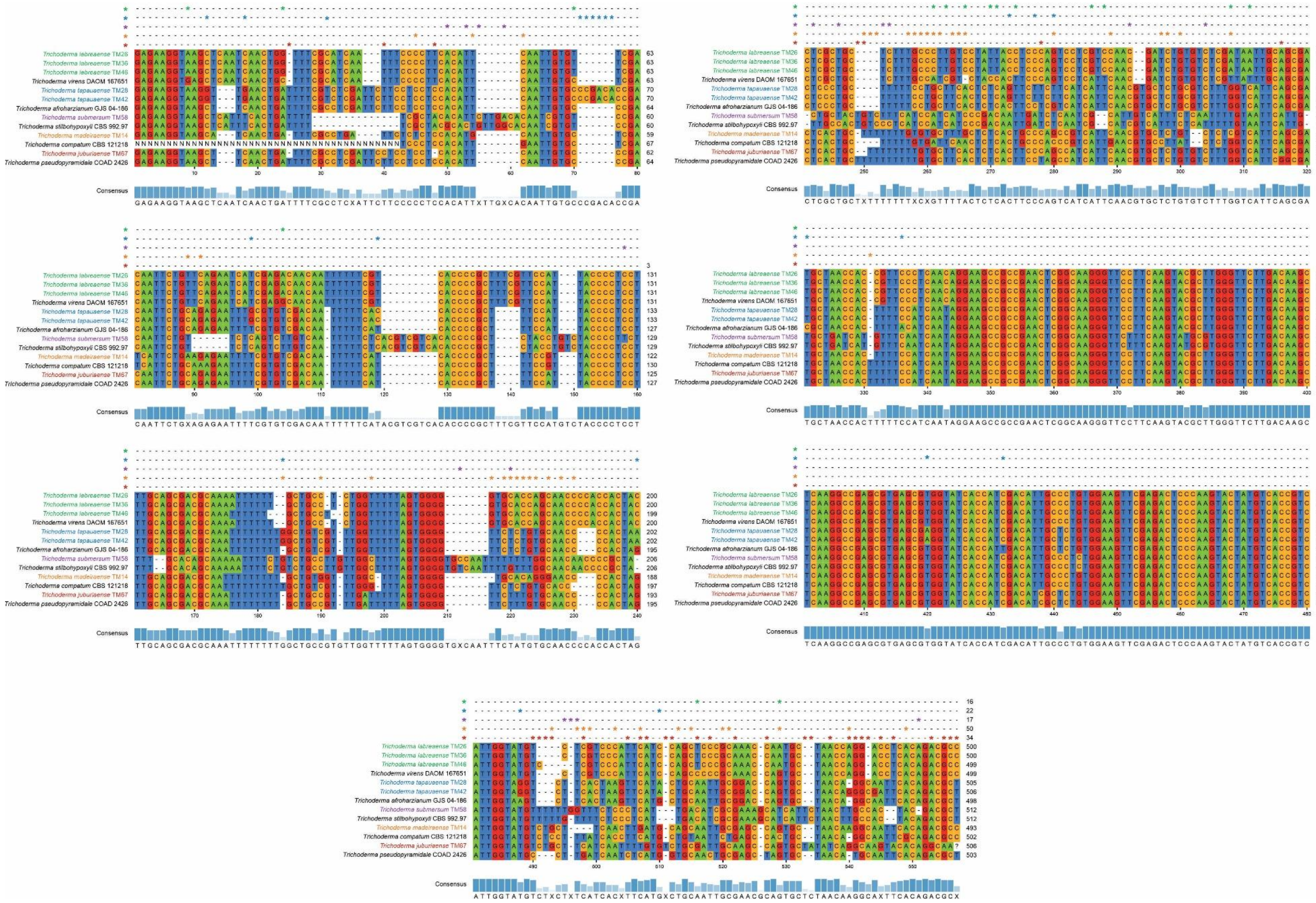

SI Figure 3.Partial *tef1-α* sequence alignment of novel *Trichoderma* species described in this study and their closest known relatives, ordered by phylogenetic affinity. Sequences are grouped by clade: *T. labreaense* clade (TM26, TM36, TM46 vs. *T. virens* DAOM 167651), *T. tapauaense* clade (TM28, TM42 vs. *T. afroharzianum* GJS 04-186), *T. submersum* clade (TM58 vs. *T. stilbohypoxyli* GJS 96-32), *T. madeiraense* clade (TM14 vs. *T. compactum* CBS 121218), and *T. juburiaense* clade (TM67 vs. *T. pseudopyramidale* COAD 2426). Diagnostic nucleotide positions, defined as sites where all isolates of a novel species share a fixed nucleotide or indel state absent in the closest reference, are indicated by color-coded asterisks (*) above the alignment, with each color corresponding to a distinct clade. Gaps (-) represent insertion/deletion events. The consensus sequence is shown at the bottom of each block.
